# Immuno-modulating properties of Tulathromycin in porcine monocyte-derived macrophages infected with porcine reproductive and respiratory syndrome virus

**DOI:** 10.1371/journal.pone.0221560

**Published:** 2019-08-23

**Authors:** D. Desmonts de Lamache, R. Moges, A. Siddiq, T. Allain, T. D. Feener, G. P. Muench, N. McKenna, R. M. Yates, A. G. Buret

**Affiliations:** 1 Department of Biological Sciences, University of Calgary, Calgary, AB, Canada; 2 Faculty of Veterinary Medicine, University of Calgary, Calgary, AB, Canada; 3 Department of Biochemistry and Molecular Biology, University of Calgary, Calgary AB, Canada; 4 Department of Comparative Biology and Experimental Medicine, University of Calgary, Calgary, AB, Canada; University of Bern, SWITZERLAND

## Abstract

Porcine reproductive and respiratory syndrome virus (PRRSV) is a positive-stranded RNA virus that grows in macrophages and causes acute pneumonia in pigs. PRRSV causes devastating losses to the porcine industry. However, due to its high antigenic variability and poorly understood immunopathogenesis, there is currently no effective vaccine or treatment to control PRRSV infection. The common occurrence of PRRSV infection with bacterial infections as well as its inflammatory-driven pathobiology raises the question of the value of antibiotics with immunomodulating properties for the treatment of the disease it causes. The macrolide antibiotic Tulathromycin (TUL) has been found to exhibit potent anti-inflammatory and immunomodulating properties in cattle and pigs. The aim of this study was to characterize the anti-viral and immunomodulating properties of TUL in PRRSV-infected porcine macrophages. Our findings indicate that blood monocyte-derived macrophages are readily infected by PRRSV and can be used as an effective cellular model to study PRRSV pathogenesis. TUL did not change intracellular or extracellular viral titers, not did it alter viral receptors (CD163 and CD169) expression on porcine macrophages. In contrast, TUL exhibited potent immunomodulating properties, which therefore occurred in the absence of any direct antiviral effects against PRRSV. TUL had an additive effect with PRRSV on the induction of macrophage apoptosis, and inhibited virus-induced necrosis. TUL significantly attenuated PRRSV-induced macrophage pro-inflammatory signaling (CXCL-8 and mitochondrial ROS production) and prevented PRRSV inhibition of non-opsonized and opsonized phagocytic function. Together, these data demonstrate that TUL inhibits PRRSV-induced inflammatory responses in porcine macrophages and protects against the phagocytic impairment caused by the virus. Research in live pigs is warranted to assess the potential clinical benefits of this antibiotic in the context of virally induced inflammation and tissue injury.

## Introduction

Responsible for estimated losses exceeding US$600 million/year in the USA alone, porcine reproductive and respiratory syndrome (PRRS) is a devastating disease in the swine industry [1]. First identified in Europe and North America in the late 1980s [2], this syndrome is currently prevalent in most swine-producing countries [3]. Its causative agent, the porcine reproductive and respiratory syndrome virus (PRRSV), is a small enveloped positive-sense single-stranded RNA virus, member of the *Arterivirus* genus [3]. Sequence comparison between viral isolates demonstrated that PRRSV exists in at least two distinct genotypes, the European genotype (EU type or type I) commonly referred to as PRRSV-1, and the North American genotype (NA type or type II) known as PRRSV-2 [4]. PRRSV has a very narrow cell tropism, and may induce persistent asymptomatic infections [5, 6].

In its natural host, the virus targets alveolar macrophages (AM) [7, 8], and is able to infect most cells of the monocyte-macrophage lineage such as intravascular and lymph node macrophages [7, 9, 10]. These cells play a crucial role in immune surveillance, pathogen killing and adaptive immune response stimulation [11]. PRRSV impairs macrophage phagocytic and bactericidal functions, induces host cell death often resulting in an inflammatory response, and perhaps most importantly predisposes the pig to secondary infections [12–16]. Indeed, opportunistic pathogens, whether viral—swine influenza virus, pseudorabies virus- or bacterial–*Streptococcus suis*, *Bordetella brochiseptica*—potentiate PRRSV-induced pneumonia [14]. These synergistic effects promote a self-sustaining inflammatory response increasing the severity and the duration of the disease [17–21].

The common occurrence of PRRSV infection with bacterial infections combined with the lack of efficient vaccines begs the question of the value of antibiotics for the treatment of PRRS. Traditionally, antibiotic efficacy is evaluated solely based on their antimicrobial properties. However, some macrolides have been found to modulate ROS and pro-inflammatory cytokines such as CXCL-8 and IL-6, and to alter the production of lipid mediators that regulate inflammation [22–27]. These antibiotics accumulate within leukocytes at concentrations that may reach 500 times the systemic levels, which in turn allows them to be transported directly to the site of infection and confers them superior pharmacodynamics [25]. There is little evidence supporting a direct anti-viral property for macrolides, but their effects on leukocytes support the hypothesis that such macrolides may be beneficial in the context of viral infections such as PRRSV [24, 25]. In an attempt to uncover new mechanisms whereby macrolides may protect against the detrimental effects of PRRSV, the present study investigated the effects of tulathromycin in porcine monocyte-derived macrophages. Tulathromycin is a triamilide in which its 15 lactone-ring is comprised of 3 polar amine groups. It is used for the treatment and prevention of swine respiratory diseases associated with *Actinobacillus pleuropneumoniae* a gram-negative bacteria often found in PRRSV-infected pigs [14]. *A*. *pleuropneumoniae* exerts cytotoxic effects in macrophage and neutrophils and increases the production of pro-inflammatory IL-6, CXCL-8 –also known as Interleukin-8- and leukotriene B_4_, which ultimately leads to severe pulmonary tissue damage and death [28–32]. Recent studies have demonstrated that in addition to its antimicrobial effects, tulathromycin inhibits CXCL-8 and LTB_4_ production in stimulated neutrophils and macrophages [26, 30, 33]. In addition, tulathromycin promotes the apoptotic death of neutrophils and their phagocytic clearance by macrophages -a phenomenon known as efferocytosis- both crucial processes in the resolution of inflammation [26, 30, 33–35]. We hypothesized that tulathromycin may generate immunomodulatory benefits in PRRSV-infected monocyte-derived macrophages. The findings indicate that tulathromycin, in the absence of a direct anti-viral effect, is able to restore the phagocytic function and to attenuate the pro-inflammatory phenotype of PRRSV-infected monocyte-derived porcine macrophages.

## Materials and methods

### Cell line and virus strain

The African green monkey kidney cell line MARC-145 (CRL-12231) which is highly permissive to PRRSV, was used for viral passage and plaque titration assay, as validated previously [36, 37, 36] MARC-145 cells were cultivated in Dulbecco’s Modified Eagle’s Medium (DMEM; Thermo Fisher Scientific, Waltham, MA, USA) supplemented with 10% FBS (Invitrogen, Carlsbad, CA, USA) and 100 IU/mL penicillin-streptomycin (Thermo Fisher Scientific, Waltham, MA, USA). The cells were maintained at 37°C, 5% CO2 and passaged twice weekly. PRRSV-2 isolate NVSL 98–7895 (GenBank accession no. AY545985.1) was used in all experiments as previously described [37]. Viral titration was performed via plaque assay. Briefly, MARC-145 cells were seeded in 12 well plates (Costar; Sigma Aldrich, Saint-Louis, MO, USA) and grown until confluency. Once at confluency, cells were infected with PRRSV, for 1 hour in serum-free DMEM to allow attachment of viral particles. Following attachment, MARC-145 were overlaid with a solution of 2X MEM diluted 1:1 with 1.5% agarose. Infection was carried for 96h and plaques were revealed with neutral red (Sigma-Aldrich, Saint-Louis, MO, USA). Dr. R. M. Yates from University of Calgary generously provided both MARC-145 cell line and PRRSV-2 isolate NVSL 98–7895.

### Blood collection

All animal experimental practices and care were conducted according to the standards of the Canadian Council of Animal Care guidelines and approved by the University of Calgary Life and Environmental Science Animal Care Committee. Blood was collected from healthy Large White and Landrace cross 10- to 22 weeks old (15- to 60 kg) female and castrated male piglets. The animals were housed at the Veterinary Science Research Station (University of Calgary) at 22°C ± 2°C with 40% humidity, light cycles consisted of 12 hours continuous light exposure followed by 12 hours of darkness. Piglets were fed twice with the antibiotic-free feed 16% Hog Grower (Hi-Pro Feeds, Okotoks, AB, Canada), water was provided *ad libitum*. After 22 weeks, animals were euthanized and tissues made available for secondary teaching and research use. In accordance with the standards of the Canadian Council on Animal Care, pigs were euthanized by intracardiac injection with sodium pentobarbital.

### Monocyte isolation and macrophage differentiation

Monocytes were obtained and differentiated into macrophages as described previously [30]. Briefly, blood was pooled and centrifuged for 20 minutes at 1200 *x g*, 4°C in a Heraeus Megafuge 16R (Thermo Fisher Scientific, Waltham, MA, USA). The plasma was removed, and the buffy coat layer was collected into and diluted 1:1 in filter-sterilized 0.9% NaCl. Sterile polysucrose and sodium diatrizoate gradient solution (Histopaque; Sigma-Aldrich, Saint-Louis, MO, USA) was added into each tube before centrifugation for 40 minutes at 1200 *x g*, 4°C. PBMCs located at the opaque interphase were then collected, washed with sterile-filtered 2X Hank’s Balanced Salt Solution (HBSS; Thermo Fisher Scientific, Waltham, MA, USA) and centrifuged for 10 minutes at 500 *x g*, 4°C. Contaminating erythrocytes were removed by three hypotonic lysis cycles with sterile ice-cold double-distilled water for 30 seconds followed by the addition of 2X HBSS to restore tonicity. PBMCs were then resuspended in serum-free Iscove’s modified Dubelcco’s medium (IMDM; Thermo Ficher Scientific, Waltham, MA, USA) supplemented with 100 IU/mL penicillin-streptomycin. Cells were counted using a hemocytometer and viability was assessed by 0.1% trypan blue exclusion (Flow Laboratories). PBMCs purity was determined by Diff-Quick staining on cytospin slides (CytoSpin4 cytocentrifuge, Thermo Fisher Scientific, Waltham, MA, USA). The cells were then plated in tissue-culture treated 6, 12, 24 and 96 well plates (Costar; Sigma Aldrich, Saint-Louis, MO, USA) or in LabTek chamber slides (Thermo Fisher Scientific, Waltham, MA, USA) at a concentration of 1.0 x10^6^ cells/mL for two hours to allow attachment. Following adhesion, non-adherent mononuclear cells were washed with warm HBSS (37°C). Subsequent adherent monocytes were incubated for 7 days at 37°C, 5% CO_2_ in IMDM supplemented with 10% heat inactivated(HI)-pig serum (GE Healthcare, Chicago, IL, USA), 100 IU/mL penicillin-streptomycin and 15% L929 supernatant to allow for differentiation into monocyte-derived macrophages (MDMs). L929-conditionned medium is commonly used to potentiate monocytes to differentiate into homogenous populations of mature macrophages [37, 38]. Culture media was changed every 3 days. Flow cytometry was used to quantify the number of cells expressing CD163 (a known cluster of differentiation of monocytes and macrophages). More than 95% of the isolated cells expressed CD163 (S1 Fig). On day 7, as described previously [26], macrophage differentiation was monitored by microscopic morphological changes using Diff-Quick, and esterase staining, a well known feature allowing to distinguish between monocytes and mature macrophages [39]. At day 7, more than 95% of the cell preparations were differentiated macrophages (data not shown).

### Tulathromycin treatment and PRRSV infection of macrophages

Seven days-old differentiated macrophages were incubated with tulathromycin (Draxxin; Zoetis, Parsippany-Troy Hills, NJ, USA) diluted in IMDM + 10% HI-pig serum at a concentration of 0.5 mg/mL or 1 mg/mL or with vehicle control (IMDM + 10% pig serum), as established recently [26, 33]. At these concentrations and time points, the drug exhibits immunomodulating properties in bovine macrophages without inducing apoptosis [26]. Antibiotics like tulathromycin accumulate within leukocytes at concentrations that may reach >500 times the systemic levels, which in turn allows them to be transported directly to the site of infection, and hence confers them with superior pharmacodynamics [25, 40]. This phenomen is critical to the mode of action of tulathromycin. The drug concentrations used in these present experiments are consistent with this knowledge, and with previous studies that showed that tulathromycin has immunomodulating effects in bovine and porcine neutrophils and macrophages [26, 30, 33]. These recent studies have reproduced the same immunomodulating effects seen *in vitro* at these drug concentrations than when using live infected cattle and pigs given tulathromycin at the recommended therapeutic dosage [26, 30, 33]. Hence the concentrations used here reflect the physiological conditions in which the drug accumulates at high concentrations within these leukocytes. Indeed, comparison of intracellular drug concentrations in treated versus untreated animals have been published previously [40]. Using LC/ MS MS, in animals given the recommended dose of 2.5 mg/kg body weight, studies have measured the rapid and prolonged distribution of the drug into lung homogenates, pulmonary epithelial ling fluid (PELF), as well as in PELF cells. Macrophages are the major constituents of PELF cells in such preparations. Drug levels measured in PELF cells reached concentrations 565 times greater than those in plasma [40]. It is believed that this great affinity for cellular uptake may be related, in part, to the tri-basic chemical structure of the drug and the trapping of ionized drug within acidic phagolysosomes.

Macrophages were infected with PRRSV 30 minutes after TUL or vehicle treatment, or not infected (uninfected controls) and incubated for 1 h at 37°C, 5% CO_2_ to allow virus attachment and entry (Time 0; T = 0). PRRSV was diluted in serum-free DMEM to reach a multiplicity of infection (m.o.i) ranging from 0.1 to 1 depending on the experiment. Culture media was replaced by pre-warmed IMDM supplemented with 10% pig serum for all experimental groups. PRRSV infection was performed for another 2 to 48 hours depending on the experiment. All functional assays contained the following experimental groups: untreated and uninfected control (control); tulathromycin-treated (TUL); untreated and PRRSV-infected (Virus); Tulathromycin-treated and PRRSV- infected (TUL+Virus); LPS-activated; pro-apoptotic positive control (staurosporine; 3μM) (STS); or pro-necrotic positive control (0.1% Triton-X) (Trit-X) where appropriate.

### Macrophage differentiation and activation

To avoid L929 cytokine-induced polarization of macrophages, all macrophages activation experiments were performed on monocytes that were grown in L929 supernatant free. The effects of tulathromycin and PRRSV on macrophage differentiation and activation was determined via microscopic observations and cytokine quantification. MDMs were treated with tulathromycin (0.5 or 1 mg/mL) for 1 hour and infected with PRRSV (m.o.i. of 0.1) for 2, 4, 12 or 24h at 37°C, 5% CO_2_. Supernantants were collected and frozen at -80°C until processed and macrophages were stained with DiffQuick (Electron Microscopy sciences, Hatfield, PA, USA) and observed with a Nikon eclipse T300 microscope to assess morphological changes. Images were taken with a Retiga 2000x camera (Q imaging, Surrey, BC, Canada) on a Leica DMR fluorescent microscope (Leica, Wetzlar, Germany) and analyzed using ImageJ software. Individual macrophage morphology was assessed and classified as “resting” or “fibroblast-like” morphology. At least 150 macrophages per group in 3 independent experiments were assessed. Supernatants were processed to measure interleukin-8 (CXCL-8) and interleukin-10 (IL-10) concentrations using the porcine CXCL-8 Quantikine enzyme-linked immunosorbent assay (ELISA; P8000, R&D systems, Minneapolis, MN, USA) and the IL-10 Quantikine ELISA (P1000, R&D systems, Minneapolis, MN, USA) respectively. Samples were processed as per manufacturer’s instructions.

### Reactive oxygen species (ROS) production

ROS production by MDMs following tulathromycin treatment and/or PRRSV infection was monitored with the Oxiselect intracellular ROS assay kit (Cell BioLabs, San Diego, CA, USA). Experiments assessed ROS production in resting cells, as well as in a group of cells induced by lipopolysaccharide (LPS) to determine the effects of the various stimuli under basal conditions in these cells, as well as when they were activated. MDMs were infected for 2, 4, 12, or 24h (m.o.i of 0.5) or uninfected (uninfected control). The same treatments were performed on macrophage stimulated with lipopolysaccharide (1μg/mL LPS from *E*. *coli* O26:B6 (Sigma-Aldrich, Saint-Louis, MO, USA). MDMs were exposed to 2’,7’-Dichlorodihydrofluorescin diacetate (DCFH-DA) a cell-permeable fluorogenic probe oxidized to highly fluorescent 2’,7’-Dichlorodihydrofluorescein (DCF) by ROS. Fluorescence intensity, proportional to ROS levels within the cytosol was measured using a SpectraMax M2e microplate reader (Molecular Devices, San Jose, CA, USA) reading at 480 nm (excitation) and 530 nm (emission).

### Phagocytosis assays

Phagocytic capacity of MDMs was assessed using non-opsonized zymosan particles and opsonized latex beads. Non-opsonized phagocytosis was monitored using fluorescently labelled *Saccharomyces cerevisiae* zymosan A particles (Texas Red; Sigma-Aldrich, Saint-Louis, MO, USA). MDMs seeded on labtek chamber slides or on coverslips at 1x10^6^ cells/mL were infected for 2 or 12 hours (m.o.i of 0.5) or not infected (uninfected control). Following infection, experimental groups were incubated with zymosan A particles diluted in control media to a final ratio of 10:1 (zymosan:cells) for 1 hour. After exposure, extracellular zymosan A particles were washed away with warm PBS and the cells were fixed in ice-cold 80% acetone solution. Actin was stained with the Alexa Fluor 488 phalloidin antibody (Thermo Fisher Scientific, Waltham, MA, USA) and the nucleus was revealed with DAPI (Thermo Fisher Scientific, Waltham, MA, USA). Enumeration of intracellular zymosan was performed using a Leica DMR fluorescent microscope. Fc-mediated phagocytic index was measured using carboxylate-modified 3μm diameter latex or silica beads (Kisker Biotech, Steinfurt, Germany) covalently coated with BSA and human IgG (Sigma-Aldrich, Saint-Louis, MO, USA). The beads were subsequently incubated with macrophages for 45 minutes at a 10:1 (beads:cells) ratio. Following phagocytosis, extracellular beads were washed away with warm PBS and the cells were stained with DiffQuick (Electron microscopy sciences, Hatfield, PA, USA) before microscopic observations. Macrophages containing one or more zymosan particles or latex beads were considered as ‘positive cells’, the phagocytic index was calculated as the ratio of positive macrophages versus total macrophages. A minimum of 150 cells per experimental group were counted from randomly selected fields. All pictures were taken using Leica DMR fluorescent microscope with a Retiga 2000x (Q imaging, Surrey, BC, Canada) and analyzed using ImageJ software. In order to prevent any counting bias slides labellings were covered with tape prior to microscopic observations.

### Macrophage apoptosis (cell death ELISA and Annexin V)

The pro-apoptotic effects of tulathromycin and PRRSV were assessed using a cell death detection ELISA kit (Roche) according to the manufacturer’s instructions as previously described [22, 26]. Absorbance was measured using a SpectraMax M2e microplate reader (Molecular Devices, San Jose, CA, USA) set at 405nm. MDMs were incubated with tulathromycin (0.5 or 1 mg/mL) for 30 minutes and infected with PRRSV (m.o.i. of 0.1) for 2, 12 or 24h at 37°C, 5% CO_2_. Similar experimental treatments were conducted with cells stimulated with LPS (1μg/mL from *E*. *coli* O26:B6; Sigma-Aldrich, Saint-Louis, MO, USA) to assess apoptosis in activated macrophages. For all experiments, cells incubated with IMDM containing 10% HI-pig serum or staurosporine (1μM) were used as negative and positive controls respectively. Annexin V staining (Roche) was performed on the same experimental groups to further assess apoptotic cell death. Staining was performed as per manufacturer’s instructions and fluorescence was observed using a Leica DMR fluorescent microscope equipped with a HCX PL FLUOTAR x40 objective (aperture = 0.75). Images were taken at 2, 12 and 24 hours post infection (p. i) with a Retiga 2000x (Q imaging, Surrey, BC, Canada). Importantly, experiments measuring cytokines and ROS followed those in which we measured apoptosis. This allowed to adjust concentrations of tulathromycin and virus to levels at which apoptosis was not detected in the cells, and hence cell death would not be a factor in the cellular cytokine and ROS responses.

### Macrophage necrosis

Necrosis was assessed through the determination of lactate dehydrogenase (LDH) levels using a cytotoxicity detection kit (Roche). MDMs were treated with vehicle medium alone (control) or with tulathromycin (1mg/mL) for 30 minutes. Cells were then infected with PRRSV for 2, 6, 12 or 24h (m.o.i. 0.1) or supplemented with control medium, and 1% Triton X 100 in media was used as positive control. Supernatants were collected and processed following manufacturer’s instructions. A SpectraMax M2e microplate reader (Molecular Devices, San Jose, CA, USA) was used to measure LDH concentrations in each sample at 492 nm. Necrosis was expressed as the absorbance ratios of the experimental cell lysates versus absorbances from controls arbitrarily set at 1.0 (100%). All experimental groups were assessed in duplicates.

### Assessment of the anti-viral effects of tulathromycin

To assess the potential anti-viral effects of tulathromycin, extracellular and intracellular viral particles counts were monitored with plaque titration assays. For extracellular counts, supernatants were harvested at 2, 12, 24 and 48 hours p.i and incubated with confluent MARC-145 cells for 96 h as described above. For intracellular counts, macrophages were washed twice with warm (37°C) phosphate buffer saline (PBS; Sigma-Aldrich, Saint-Louis, MO, USA) and lysed with double distilled water exposure and thorough mixing. Cellular debris were spun down at 10,000 *x g* for 30 minutes and supernatants were harvested and incubated with confluent MARC-145 cells as described previously. PRRSV staining was performed to further characterize potential anti-viral effects. MARC-145 cells were seeded in LabTek chamber slides, grown to 90% confluence and infected with PRRSV (m.o.i. 0.1) for 24h at 37°C, 5% CO_2_. PRRSV foci numbers and size were revealed using the SR-30F antibody (RTI, LLC, Brooking, SD, USA). Fluorescence ratio was calculated using ImageJ. Five fields of view per well were counted per sample.

### Staining of PRRSV attachment receptors

Expression levels of PRRSV receptors in MDMs in the presence and absence of tulathromycin were assessed by immunofluorescence. Seven days old MDMs cultivated with or without L929 conditioned medium were treated with HBSS (control) or tulathromycin (1 mg/mL for 12 hours). The murine L929 fibroblast cell line, known to secrete macrophage-colony stimulating factor (M-CSF) is widely used to induce macrophage differentiation from monocytes and prevent differentiation into monocyte-derived dendritic cells [38]. Prior to staining, L929-grown cells were washed 3 times in ice-cold PBS to remove all L929 media and then fixed in 4% paraformaldehyde in PBS for 15 minutes. Fixed cells were then washed 3 times in cold PBS and stained for 1 hour with a R-phycoerythrin (RPE) conjugated anti-CD163 antibody (Bio-Rad, Hercules, CA, USA) and a fluorescein isothiocyanate (FITC) conjugated anti-CD169 antibody (Bio-Rad, Hercules, CA, USA) at a dilution of 1 to 500 and 1 to 250 respectively. Following staining cells were washed 3 times in cold PBS and observed under Leica DMR fluorescent microscopy. Fluorescence ratios from randomly selected fields were calculated using the software ImageJ. Images were taken with a Retiga 2000x camera. To prevent any bias, slide labelling was covered with tape prior to microscopic observations.

### Statistical analysis

All statistical analyses were made using Prism 5 software and data were expressed as means + standard error from mean (SEM). All data sets were tested for normality. Data with parametric distribution were compared using student’s *t*-test, or one-way ANOVA with Tukey’s multiple comparision anlaysis where appropriate. Non-parametric data were compared with a Kruskal-Wallis test. For every assay, a minimum of 3 separate, independent experiments were conducted with all experimental groups assayed in duplicates or triplicates. Statistical significance was established at *P* < 0.05.

## Results

### Blood monocyte derived macrophages (MDMs) are reproducibly infected by PRRSV

**Fig 1:**
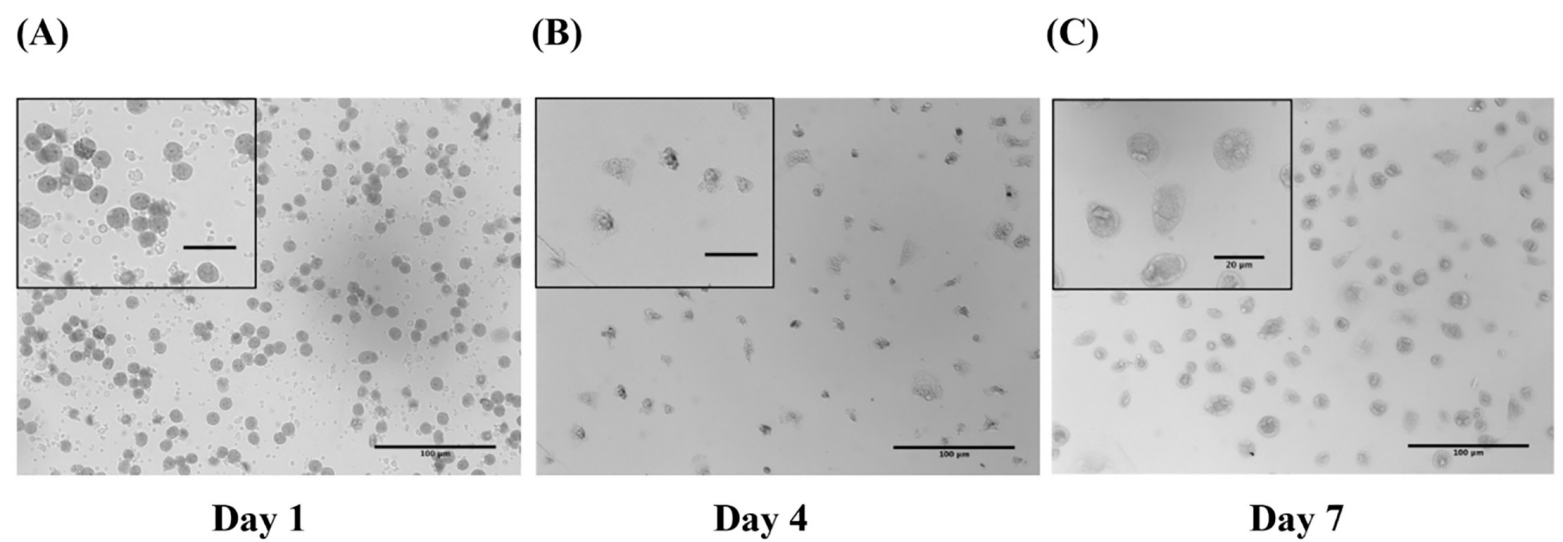
L929 cell supernatant differentiates porcine peripheral blood monocytes into macrophages *in vitro*. Cells cultured for (A) 1 (B) 4 and (C) 7 days following seeding onto plastic 24 and 48 well plate or cover slides were stained using Diff Quick. On day 7, cells are chararcterized by large macrophage-like morphology, forming typical cytoplasmic vacuolar structures and extensive pseudopods. Bar = 100 μm, bars within the insert = 20μm.

**Fig 2:**
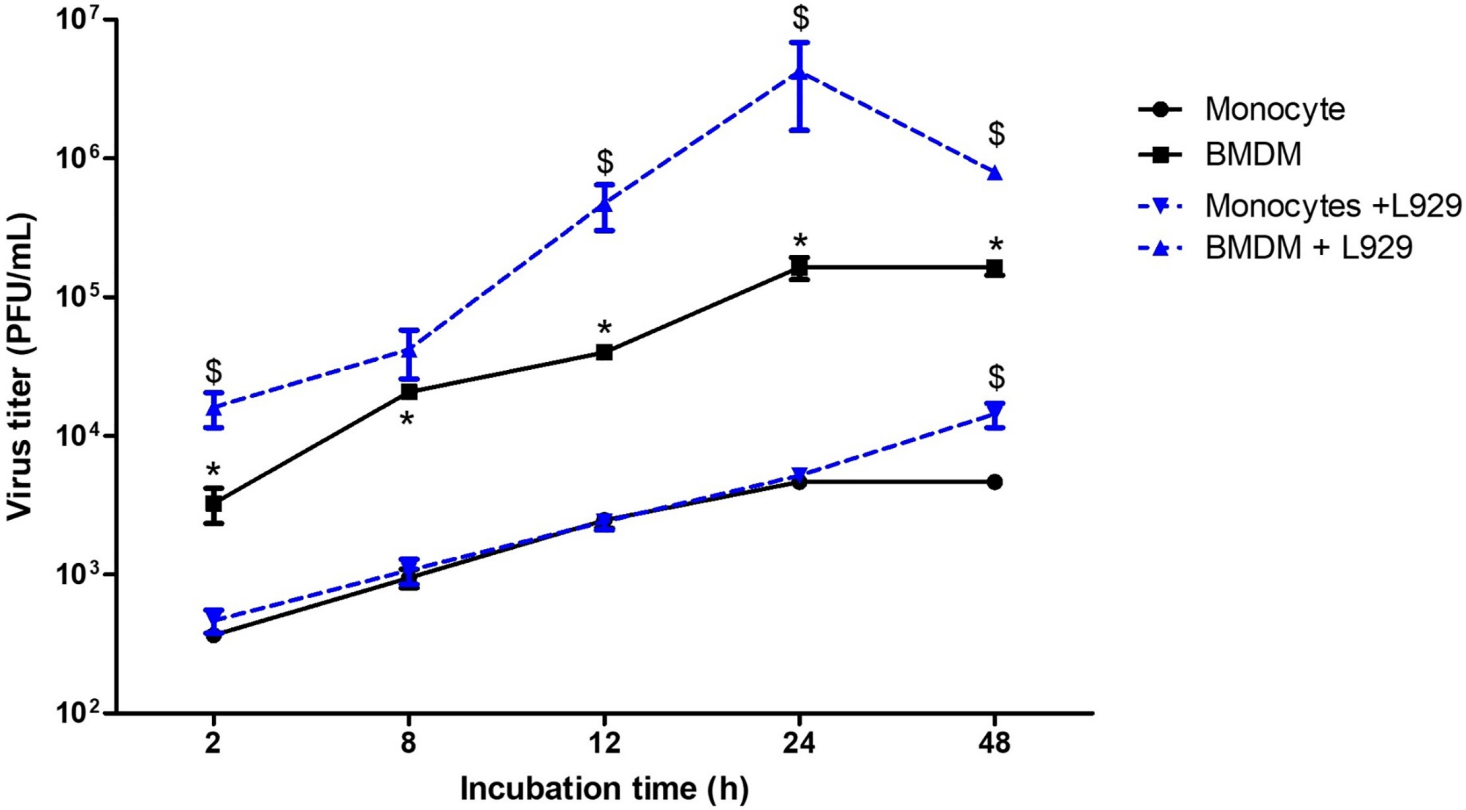
PRRSV infects porcine blood monocyte-derived macrophages more efficiently than monocytes, and infectivity is increased by L929 supernatant. Virus titers in monocytes and differentiated macrophages (MDM) infected at an m.o.i. of 0.1 over time, in presence (+ L929) or absence of L929 supernatants. Cells were lysed and viral titers calculated from supernatants by plaque assay on confluent MARC-145 cells. Data are representative of at least 3 independent experiments, and supernantants were titrated in technical duplicates. Data are expressed as mean ± SEM; * *P*<0.05 vs monocytes; $ *P* < 0.05 L929-treated vs untreated MDM or monocytes respectively.

In order to determine whether blood monocytes and MDMs are susceptible to PRRSV we isolated blood monocytes from healthy pigs and cultured them for a period of 7 days in medium supplemented with pig serum to mimic biological conditions. After plating, adherent monocytes exhibited a round shape morphology and were approximatively 10μm in diameter (Fig 1A). By day 7, the cells displayed a larger, macrophage-like, morphology with characterisitic cytoplasmic vacuoles (Fig 1C). Monocyte differentiation was also measured using non-specific esterase (NSE) staining. By day 7 more than 95% cells were esterase-positive cells. One-day-old monocytes were significantly less susceptible to PRRSV compared to 7 days old differentiated MDMs (Fig 2). PRRSV viral particle numbers increased by a 1.69 log (50-fold increase) in MDMs, and by a 1.1 log (13-fold increase) in blood monocytes, between 2 and 48 hours p.i. In both cell types, PRRSV infection reached a plateau at 24h p.i. (Fig 2).

### L929 supernatant increases PRRSV infectivity in MDMs

To optimize the macrophage differentiation protocol, MDMs were also cultured in a L929-conditioned medium. Monocytes cultivated in L929-conditioned medium showed the same morphology as those cultivated in medium devoid of L929-factors. Consistent with previous data, microscopic observation and NSE staining showed that by day 7, more than 95% cells were macrophages. Viral titers in monocytes incubated with L929 were significantly higher at 48 hours p.i. compared to viral titers in monocytes cultivated without L929 supernatant (Fig 2). Numbers of PRRSV infectious particles were significantly elevated in MDMs cultivated with L929-supernatants at all time points of the infection (except from the 8 hours p.i. time point) versus MDMs cultivated in medium supplemented with HI-pig serum alone (Fig 2). In L929-cultivated MDMs, PRRSV infection peaked at 24 h p.i. and declined afterwards, whereas it continued to increase at 48 hours in L929-cultivated monocytes (Fig 2). Based on these observations, we chose to use L929-cultivated MDMs for functional experiments, unless stated otherwise.

### Tulathromycin prevents PRRSV-induced MDMs morphological alterations

**Fig 3:**
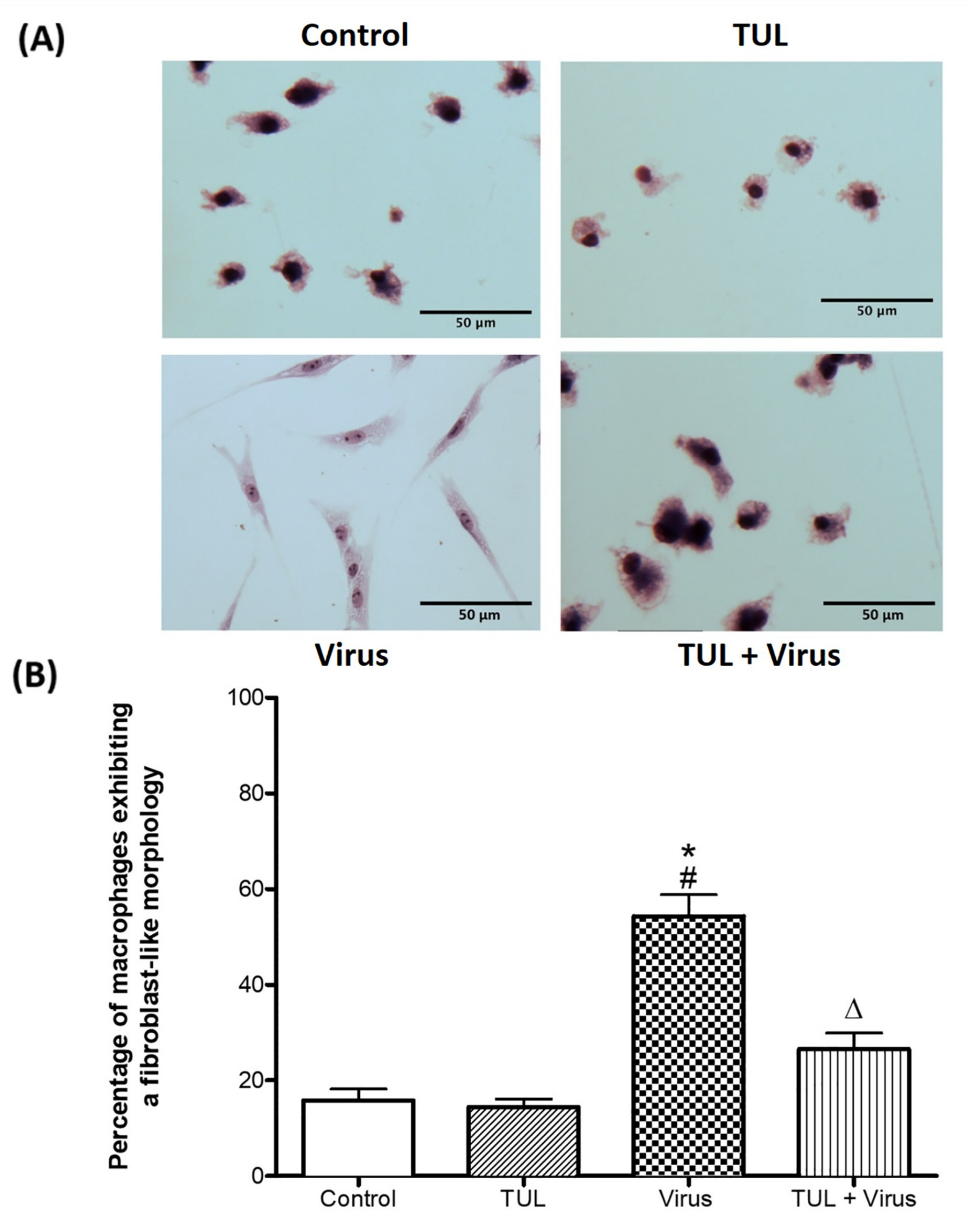
Tulathromycin prevents PRRSV-induced morphological alterations of porcine MDMs. Seven days-old porcine macrophages treated with HBSS (control) or tulathromycin (0.5 mg/mL; TUL) for 1h, un-infected or infected with with PRRSV at an m.o.i. of 0.1 for 12 hours (Virus and TUL+ Virus). (A) Staining of cells by Diff Quick reveals that infection with PRRSV alters macrophage morphology by giving them a fibroblast-like appearance (“virus”). TUL treatment prevented these alterations (“TUL+Virus”). Representative microscopic images of 5 independent experiments; Bar = 50μm. (B) Quantitative illustration of the observations shown in A, calculated as percentage of macrophages exhibiting morphological changes. At least 150 macrophages were counted for each experimental group. Data represent mean ± SEM, n = 5 per group. # = P<0.05 vs control; * = P<0.05 vs TUL; Δ = P<0.05 vs virus.

Considering that L929 supernatants may contain cytokines other than M-CSF (such as IL-10 and IL-4) that could influence macrophage polarization and confound our studies, we decided to grow our cells in medium containing only pig serum when assessing macrophage pro-inflammatory signaling. Moreover, to limit the impact of tulathromycin and PRRSV-induced apoptosis on macrophage numbers and functions, we treated our cells with tulathromycin at a concentration of 0.5 mg/mL and decreased PRRSV m.o.i from 0.5 to 0.1. At these concentrations, neither the virus nor the drugs significantly induced MDM apoptosis at the experimental time points (data not shown). If the cells were treated at a concentration of 1mg/mL, functional analysis were performed before 12 hours of incubation. PRRSV infection induced a sharp fibroblast-like morphological alteration and pseudopod projections in MDMs (Fig 3A). In uninfected cells (control and TUL), less than 20% of cells exhibited this change in morphology, while nearly 60% of the cells exhibited this phenotype upon PRRSV infection (Fig 3B). Tulathromycin pre-treatment significantly inhibited this morphological change in MDMs (Fig 3B). Since macrophage shape and function are correlated [41], we hypothesized that PRRSV-induced morphological changes were associated with a change in macrophage activation.

### Tulathromycin inhibits PRRSV-induced CXCL-8 secretion

**Fig 4:**
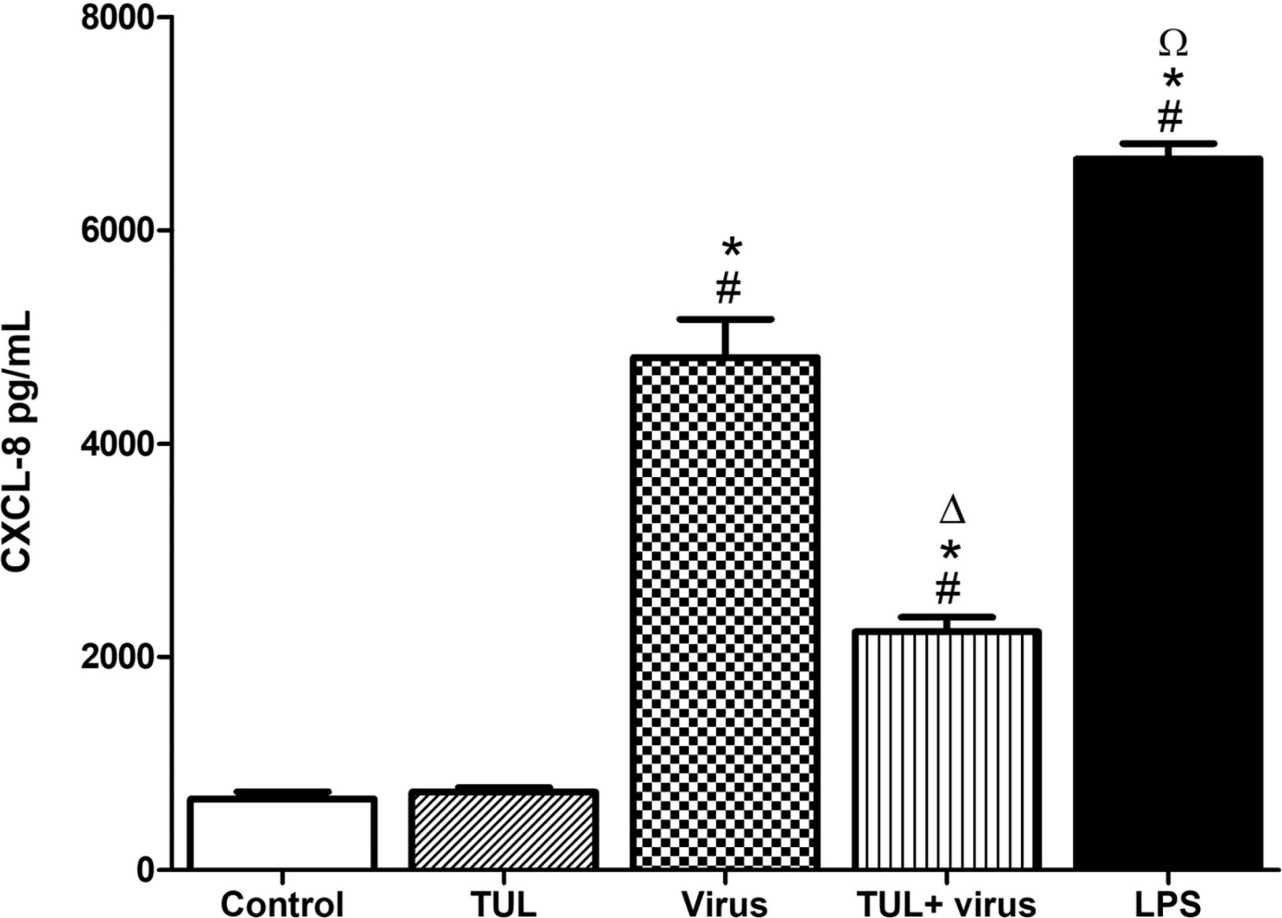
Tulathromycin inhibits PRRSV-induced CXCL-8 secretion in porcine MDMs. CXCL-8 secretion in 7 days old MDMs following HBSS (control) or TUL treatment (0.5mg/mL; TUL) for 1 hour, un-infected or infected with PRRSV infection (m.o.i = 0.1; Virus and TUL+Virus) for 24 hours measured by ELISA. Lipopolysaccharides (LPS; 1μg/mL) served as a positive control. Mean ± SEM, n = 4 per group. # = P<0.05 vs control; * = P<0.05 vs tulathromycin; Δ = P<0.05 vs virus; Ω = P<0.05 vs TUL + virus.

Infection of MDMs with PRRSV caused a 6—fold increase of CXCL-8 secretion after 24 hours (Fig 4). PRRSV-induced CXCL-8 secretion was significantly inhibited when MDMs were pre-treated with tulathromycin (Fig 4). The positive control LPS, also significantly increased the production of CXCL-8 (Fig 4).

### Tulathromycin inhibits intracellular ROS production

**Fig 5:**
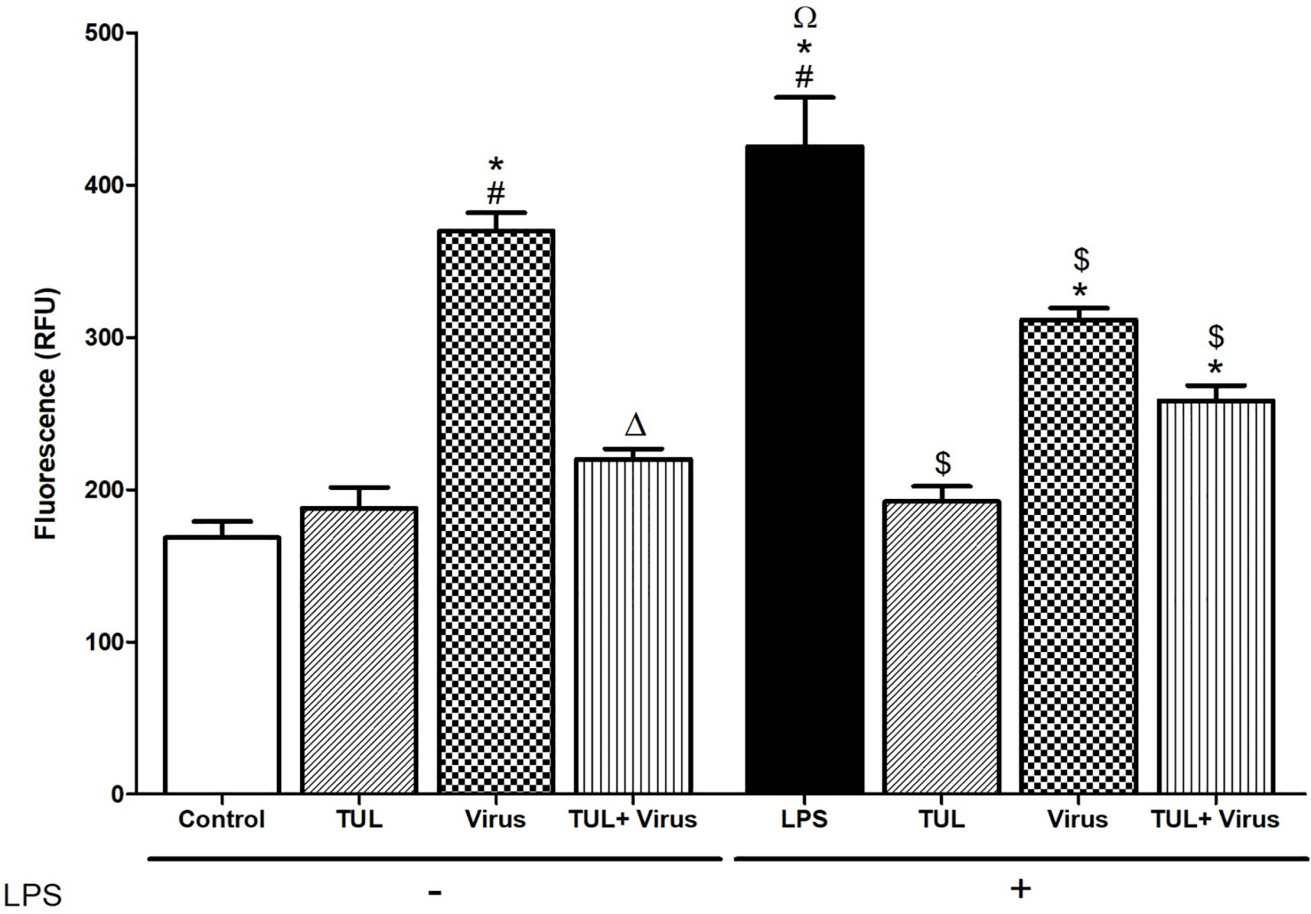
Tulathromycin inhibits PRRSV-induced mitochondrial reactive oxygen species production in porcine MDMs. Levels of intracellular ROS in resting MDMs (left panel) and in lipopolysaccharides (LPS)-activated MDMs (right panel). 7 days old MDMs were exposed to HBSS (control) or tulathromycin (0.5 mg/mL; TUL) for 1 hour and then left un-infected or infected with PRRSV (m.o.i. = 0.1; Virus and TUL+Virus) for 24 hours. To measure intracellular ROS in LPS-activated MDMs, cells were exposed to LPS (1μg/mL) for 1 hour before incubation with TUL and PRRSV.Mean ± SEM, n = 4 per group. # = P<0.05 vs control; * = P<0.05 vs TUL; Δ = P<0.05 vs virus; Ω = P<0.05 vs TUL + virus; $ = P<0.05 vs LPS.

We then measured the production of mitochondrial ROS, a hallmark of pathogenic oxidative damage in inflamed tissues [42, 43]. PRRSV infection significantly increased intracellular ROS in resting and LPS-activated MDMs versus controls (Fig 5). Tulathromycin treatment abolished PRRSV and LPS-induced intracellular ROS production, however, it did notrestore ROS levels to control values in cells exposed to both LPS and PRRSV (Fig 5). Interestingly, intracellular ROS levels were significantly lower in MDMs incubated with LPS and PRRSV compared to MDMs stimulated only with LPS (Fig 5).

### Tulathromycin prevents PRRSV inhibition of IL-10

**Fig 6:**
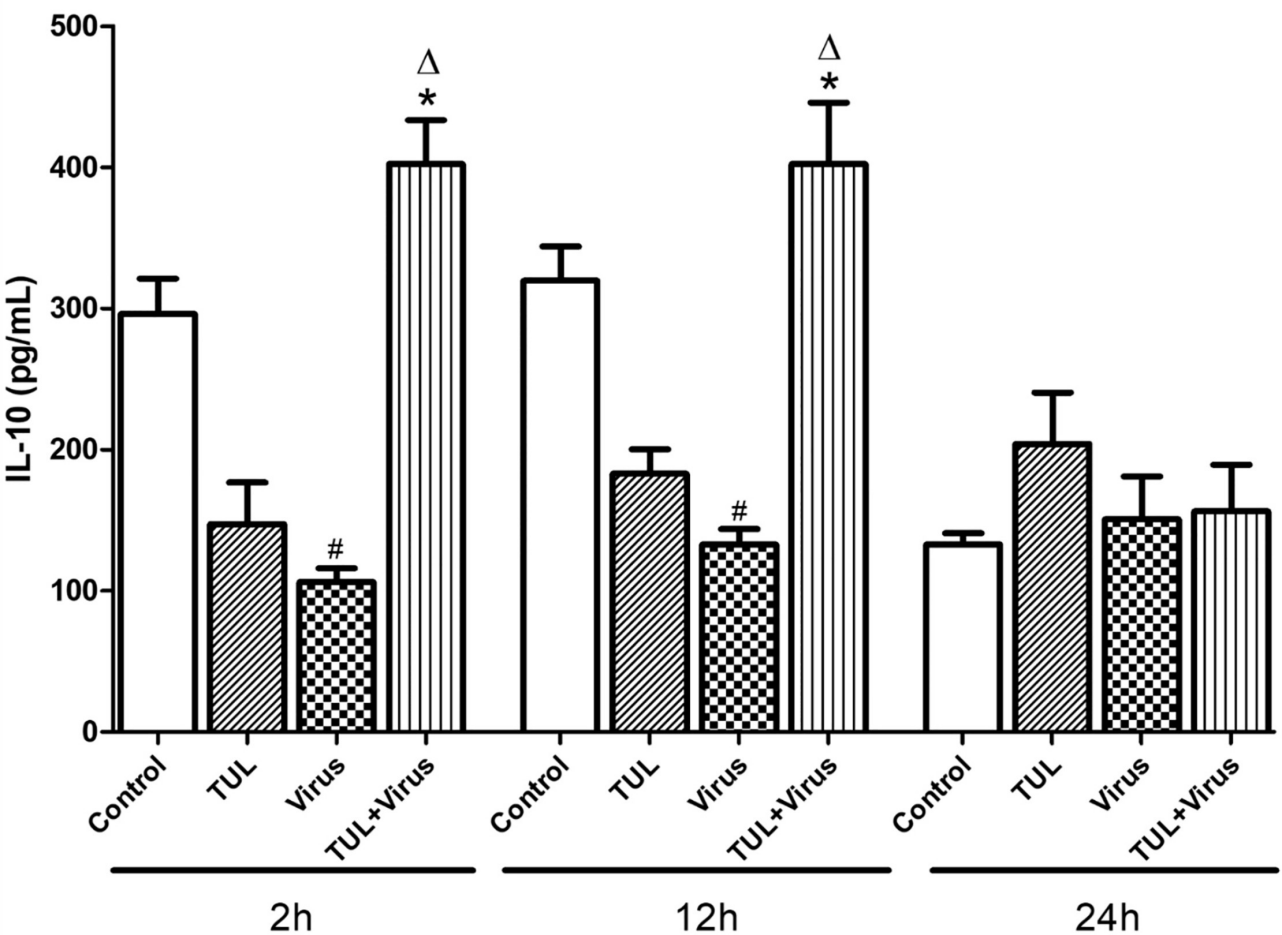
Tulathromycin prevents PRRSV-inhibition of IL-10 secretion in porcine MDMs. Levels of secreted interleukin-10 by 7 days old MDMs following HBSS (Control) or tulathromycin treatment (0.5mg/mL; TUL), un-infected or infected with PRRSV (m.o.i. = 0.1, virus and TUL+ Virus) at 2h, 12h, and 24 h post-incubation. Mean ± SEM, n = 3 per group. # = P<0.05 vs control; * = P<0.05 vs TUL; Δ = P<0.05 vs virus.

As our results indicated that TUL inhibited macrophage pro-inflammatory signaling, another set of experiment assessed the effects of the drug on IL-10, a cytokine with potent anti-inflammatory properties [44]. Resting MDMs produced approximately 300 pg/mL IL-10 throughout the course of the experiments (Fig 6). PRRSV infected cells secreted significantly less IL-10 compared to control cells at 2 and 12 hours p.i. (Fig 6). IL-10 levels did not significantly change versus controls when uninfected cells were treated with tulathromycin alone. However, PRRSV-induced IL-10 inhibition was abolished when the cells were pre-treated with tulathromycin at 2 and 12 hours post infection (Fig 6).

### Tulathromycin and PRRSV exhibit an additive effect on the induction of apoptosis in MDMs

**Fig 7:**
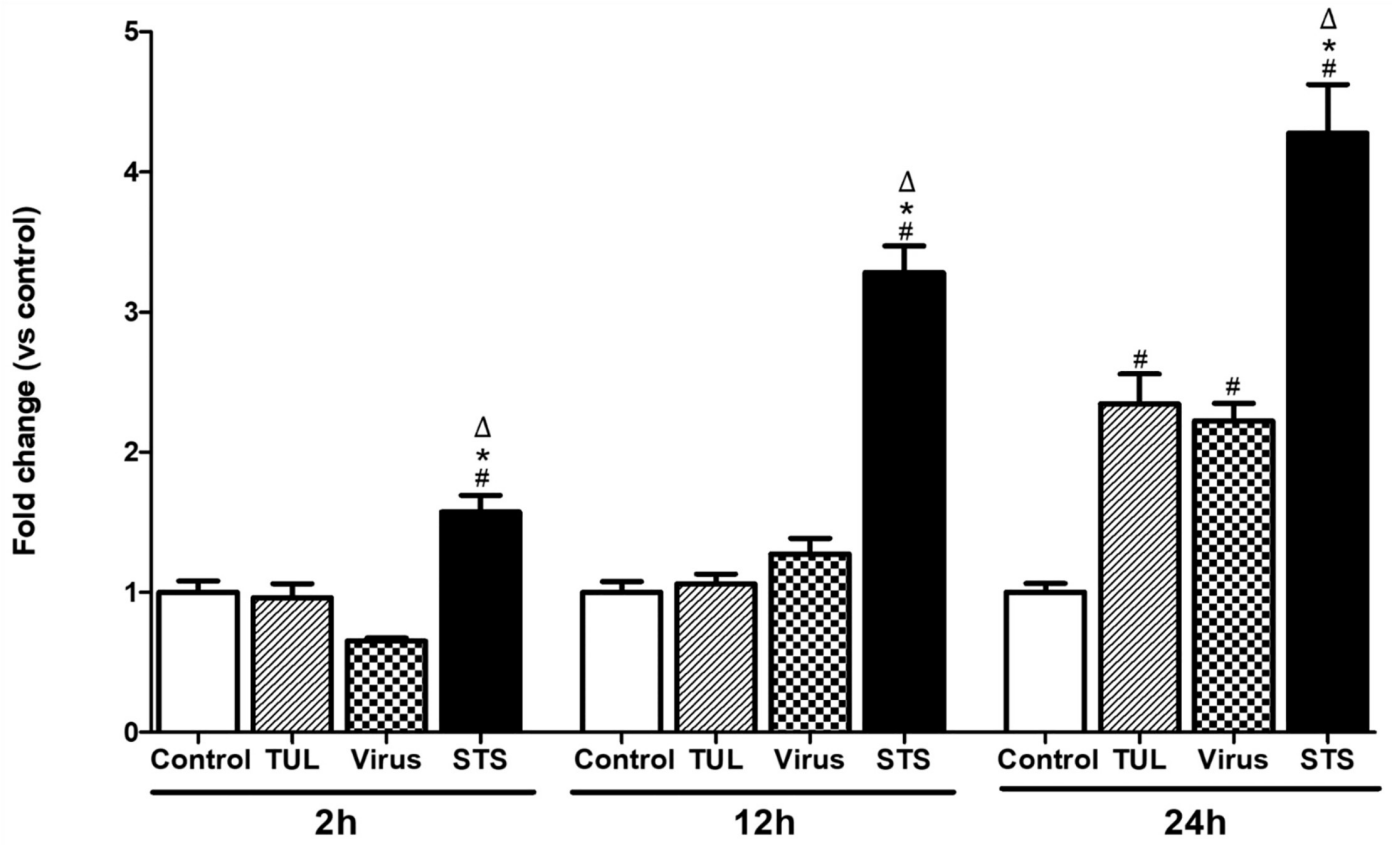
Tulathromycin and PRRSV induce time dependent apoptosis in porcine MDMs. Cell apoptotic death as detected by cell death ELISA in MDMs treated with HBSS (control) or tulathromycin (1mg/mL; TUL) for 1 hour, un-infected or infected with PRRSV (m.o.i = 0.5; Virus or TUL + Virus) at 2h, 12h, or 24h post-incubation. Staurosporine (STS) served as positive pro-apoptotic control. Values are ratios vs control, expressed as Mean ± SEM n = 4–5 per group. # = P<0.05 vs. control; * = P<0.05 vs TUL; Δ = P<0.05 vs virus.

**Fig 8:**
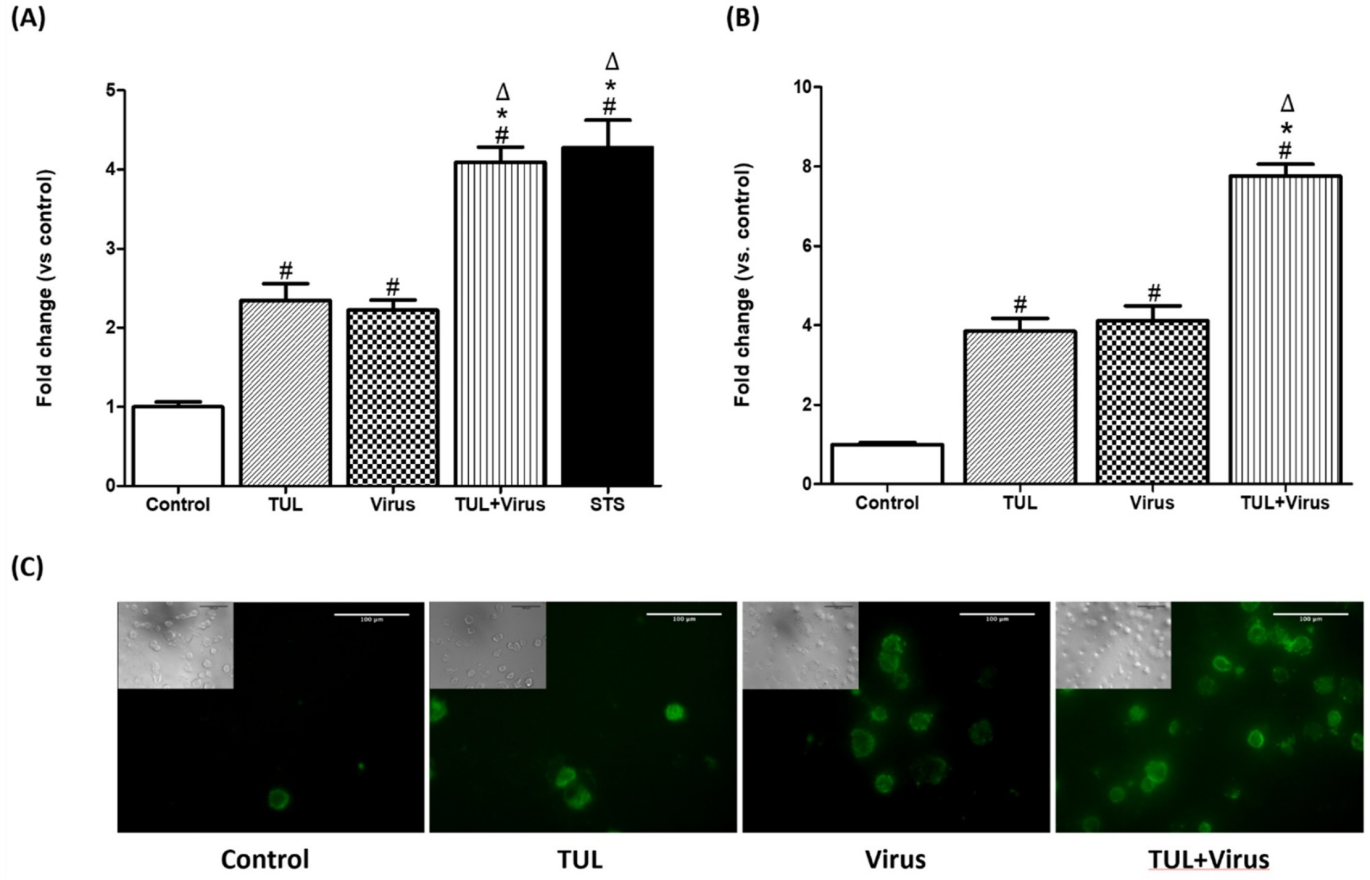
Tulathromycin PRRSV exhibit an additive effect on the induction of apoptosis in MDMs. Cell apoptotic death as detected by (A) cell death ELISA or (B, C) AnnexinV FLUOS staining in MDMs treated with HBSS (Control) or tulathromycin (1mg/mL; 1 hour; TUL), un-infected or infected with PRRSV (m.o.i = 0.5; 24 hours; virus and TUL+ Virus). Staurosporine (STS) served as a positive pro-apoptotic control. Values are expressed as (A) absorbance and (B) fluorescence ratios vs control. (A) Data represent mean ± SEM, n = 4 per group. # = P<0.05 vs control; * = P<0.05 vs tulathromycin; Δ = P<0.05 vs virus. (B) Data represent mean ± SEM, n = 5–7 per group. # = P<0.05 vs control; * = P<0.05 vs tulathromycin; Δ = P<0.05 vs virus. Pictures in panel (C) are representatives of 3 independent experiments.

Tulathromycin (1 mg/mL), as well as PRRSV alone (m.o.i = 0.5), or the positive control staurosporine induced MDMs apoptosis 24 h post-infection (Fig 7). Combined pre-treatment with tulathromycin (1mg/mL; 1h) and PRRSV (m.o.i = 0.5) for 24 hours showed an additive effect to induce further MDMs apoptosis versus single treatments (Fig 8A). To confirm these data, cells were stained with annexin V, a phospholipid-binding protein with high affinity for the early apotptic marker phosphatidylserine (PS) [45]. At 24 hours, both tulathromycin alone or PRRSV alone induced significant levels of apoptosis compared to controls (4—fold increase vs. control) (Fig 8B and 8C). When cells were exposed to the combination of tulathromycin and PRRSV, levels of apotosis were almost double those measured in cells exposed to single treatments (Fig 8B and 8C).

### Tulathromycin prevents PRRSV-induced early necrosis

**Fig 9:**
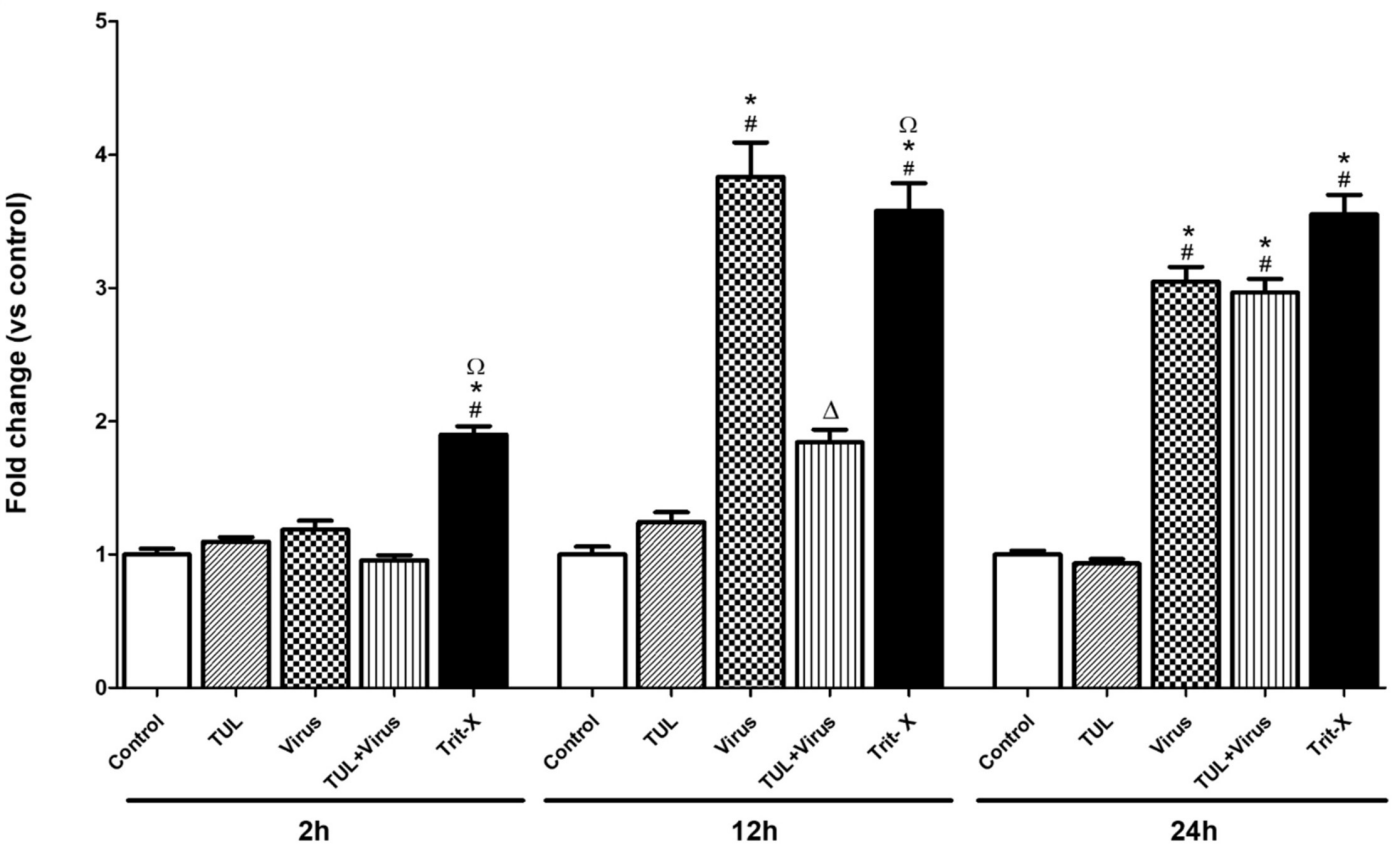
Tulathromycin prevents PRRSV-induced necrosis in porcine MDMs (at 12h). Levels of macrophages necrosis measured using a lactate dehydrogenase assay in cells incubated with HBSS, (control) or with tulathromycin (1mg/mL; TUL) for 1 hours, un-infected or infected with PRRSV (m.o.i = 0.5; virus and TUL+virus) for 2h, 12h, or 24h post-incubation. Triton X served as positive pro-necrotic control. Values are expressed as ratios vs control. Data represent mean ± SEM, n = 5 per group. # = P<0.05 vs control; * = P<0.05 vs TUL; Δ = P<0.05 vs virus; Ω = P<0.05 vs TUL + virus.

PRRSV infection (m.o.i = 0.5) significantly increased the levels of LDH produced during necrosis 12 and 24 hours p.i. (Fig 9). Treatment with tulathromycin (1mg/mL) significantly reduced PRRSV cell necrosis at 12 hours (Fig 9). This effect of tulathromycin could no longer be detected at 24 hours. Tulathromycin alone did not alter levels of necrosis (Fig 9). Triton-X (Trit-X), used as a pro-necrotic positive control, induced necrosis in MDMs (Fig 9).

### Tulathromycin restores non-opsnonized and opsonized phagocytosis of infected MDMs

**Fig 10:**
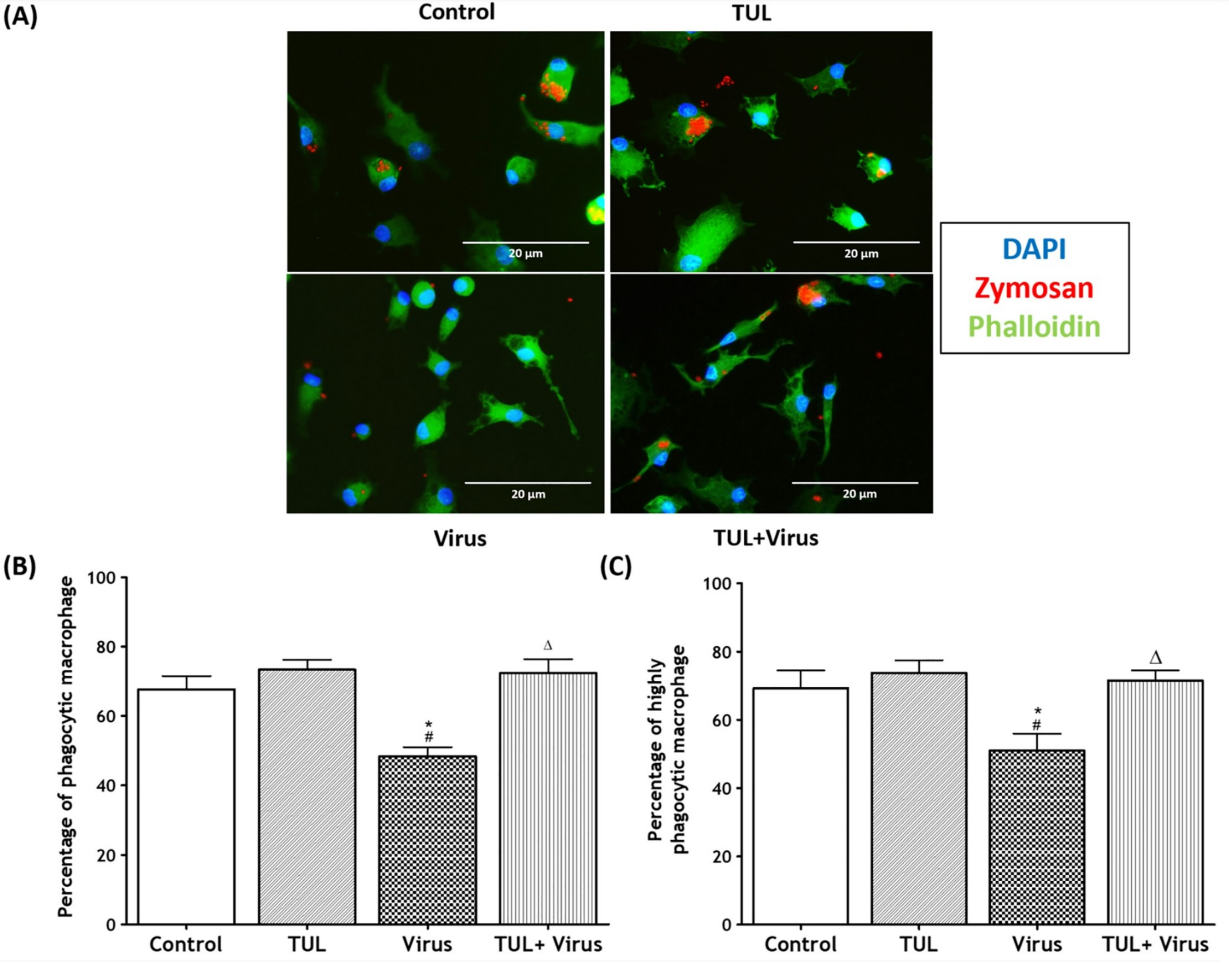
Tulathromycin restores non-opsonized phagocytosis of PRRSV-infected porcine MDMs. Non-opsonized phagocytic uptake of fluorescently labeled zymosan particles in MDMs treated with HBSS (Control) or with tulathromycin (1mg/mL; TUL) for 1h, un-infected or infected with PRRSV (m.o.i. = 0.5; Virus and TUL+Virus) for 12 hours. Following treatment and infection, MDMs were incubated with fluorescently labeled zymosan particles (10:1 ratio; Zymosan: MDMs) for 1h. (A) Representative laser confocal micrographs of porcine MDMs (red, phagocytosed zymosan particles; blue, nuclei (stained with DAPI); green actin (stained with phalloidin). (B) Percentage of macrophages that phagocytosed at least 1 zymosan particle. (C) Percentage of macrophages that phagocytosed at least 5 zymosan particles. n = 150–300 macrophages/group. Images and histograms are representative of 5 independent experiments. Mean ± SEM. # = P<0.05 vs control; * = P<0.05 vs TUL; Δ = P<0.05 vs virus.

**Fig 11:**
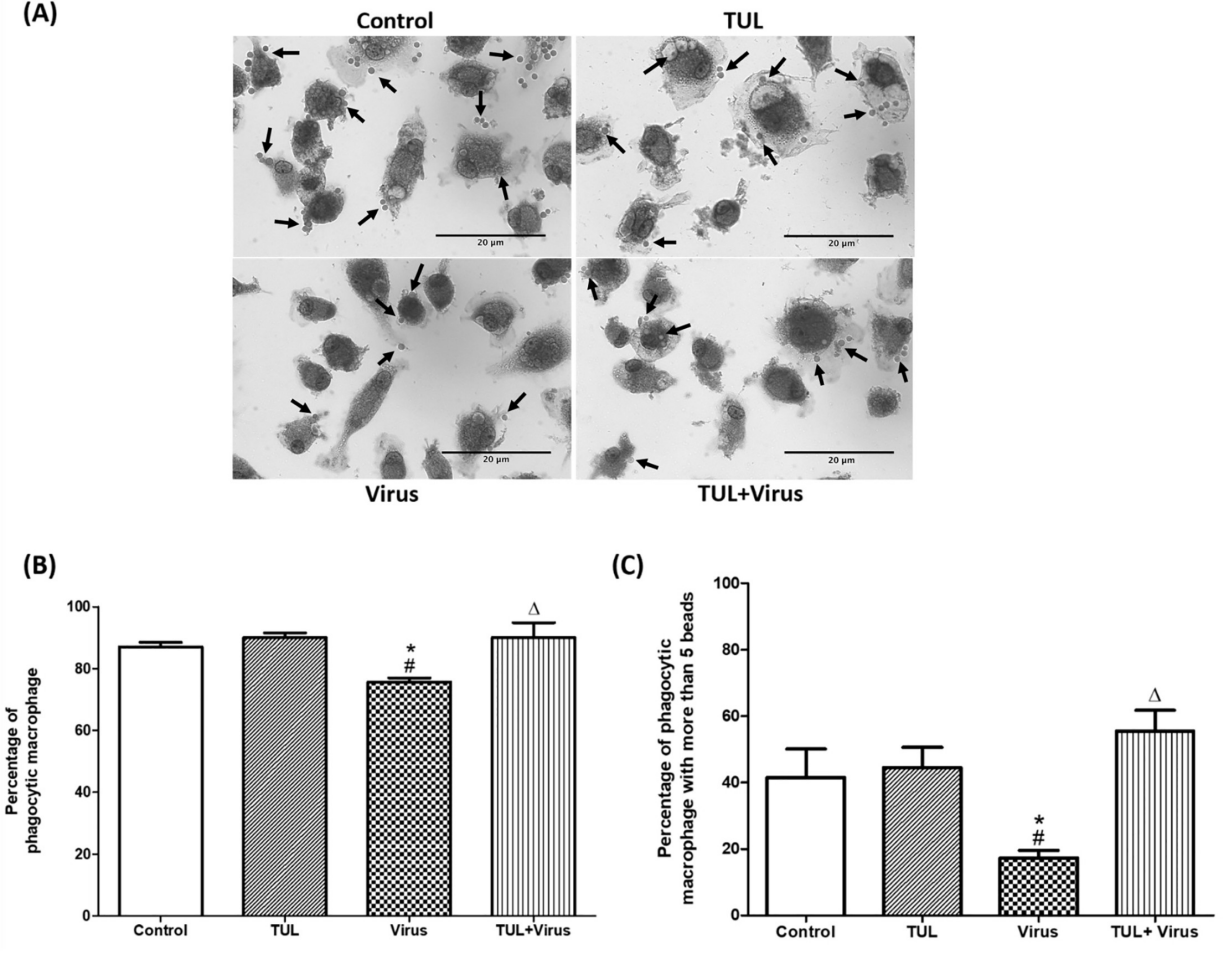
Tulathromycin restores Fc-mediated phagocytosis in PRRSV-infected porcine MDMs. Opsonized phagocytosis of IgG coated latex beads in MDMs following HBSS (control) or tulathromycin (1mg/mL; TUL) treatment for 1h and infection with PRRSV (m.o.i. = 0.5; Virus and TUL+Virus) for 12 hours was measured. Following treatment and infection, MDMs were incubated with IgG coated latex beads for 45 minutes. (A) Micrographs of macrophages that have phagocytosed latex beads (arrows). Bar = 20μm (B) Percentage of macrophages that phagocytosed at least 1 latex bead. (C) Percentage of macrophages that phagocytosed at least 5 IgG-coated latex beads. n = 150–200 macrophages/group. Images and histograms are representative of 4 independent experiments. Mean ± SEM. # = P<0.05 vs Control; * = P<0.05 vs TUL; Δ = P<0.05 vs virus.

**Fig 12:**
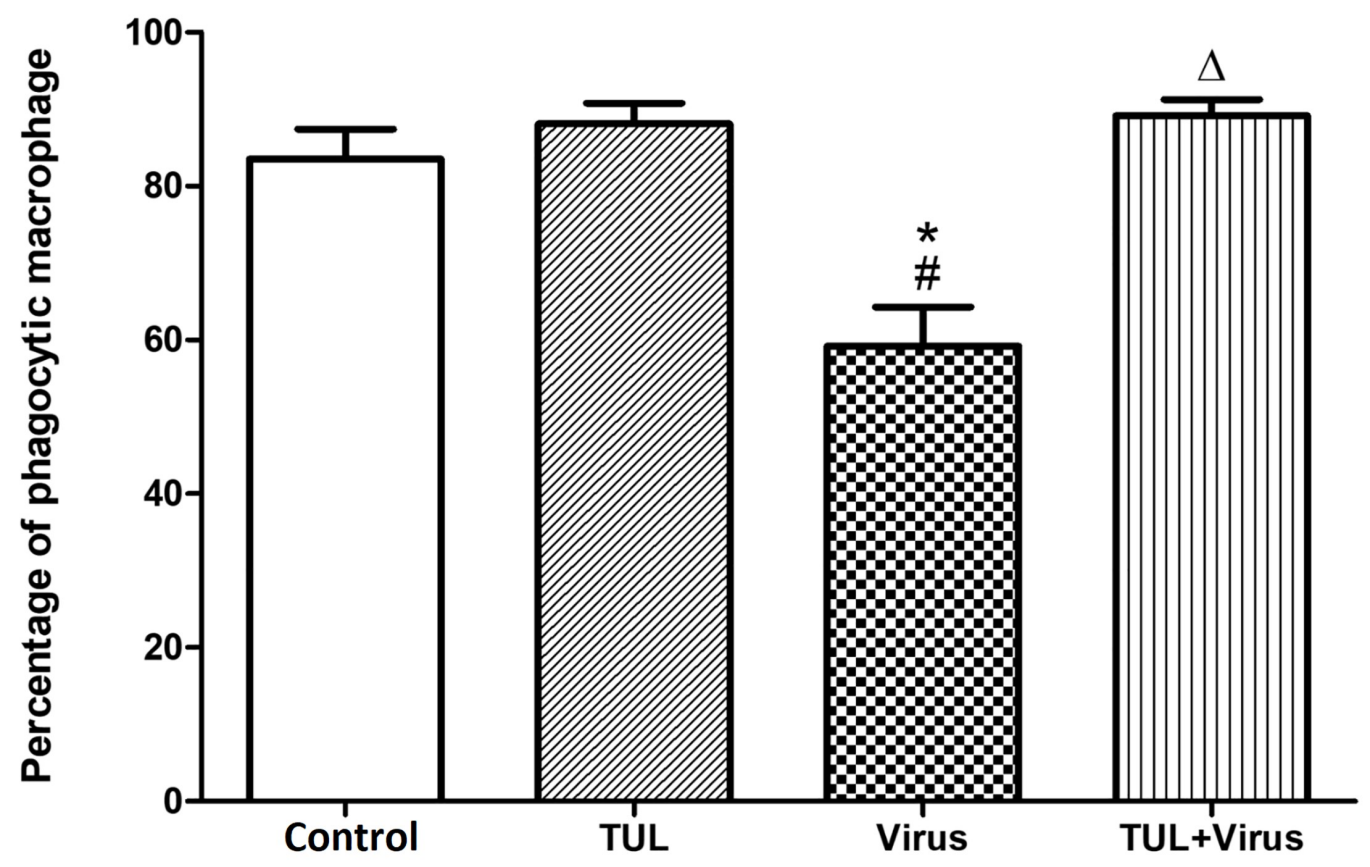
PRRSV replication is not necessary to inhibit phagocytosis in porcine MDMs. To measure the effects of PRRSV infection and tulathromycin on early phagocytosis, 7 days-old MDMs were treated with HBSS (Control) or with tulathromycin (1 mg/mL; TUL) for 1h at 37°C. and then infected with PRRSV (m.o.i. = 0.5; Virus and TUL+Virus) for 2 hours at 37°C. Then, MDMs were incubated with IgG coated latex beads (10:1 ratio; Zymosan: MDMs) for 45 minutes at 37°C. Cells were washed 3 times with warm PBS to remove free beads Percentage of macrophages that phagocytosed at least 1 IgG coated latex bead. n = 150–200 macrophages/group. Histograms are representative of 3 independent experiments. Mean ± SEM. # = P<0.05 vs control;* = P<0.05 vs tulathromycin; Δ = P<0.05 vs virus.

Another set of experiments assessed the effects of PRRSV, and of tulathromycin, on the non-opsonized and opsonized phagocytic functions of MDMs. PRRSV infection significantly inhibited both phagocytic functions of the cells (Figs 10 and 11). PRRSV-induced phagocytic inhibition was inhibited by tulathromycin (Figs 10B and 11B). Tulathromycin treatment alone did not alter MDMs phagocytosis versus controls (Figs 10 and 11). During phagocytosis, macrophages can engulf multiple antigens at the same time [46, 47]. Additional experiments assessed MDMs that engulfed less than 5 particles (ie with basal phagocytic indices) versus cells that ingested more than 5 particles (i.e. with elevated phagocytic indices). PRRSV infection significantly reduced the number of cells with high phagocytic indeces, an effect that was abolished by tulathromycin (Figs 10C and 11C). Tulathromycin inhibited the PRRSV-induced reduction of basal and high phagocytic indices (Figs 10 and 11). Tulathromycin alone did not change either of the MDMs phagocytic indices versus controls. The same results were obtained when the cells were infected for 2 hours (Fig 12).

### Tulathromycin does not alter PRRSV viral counts

**Fig 13:**
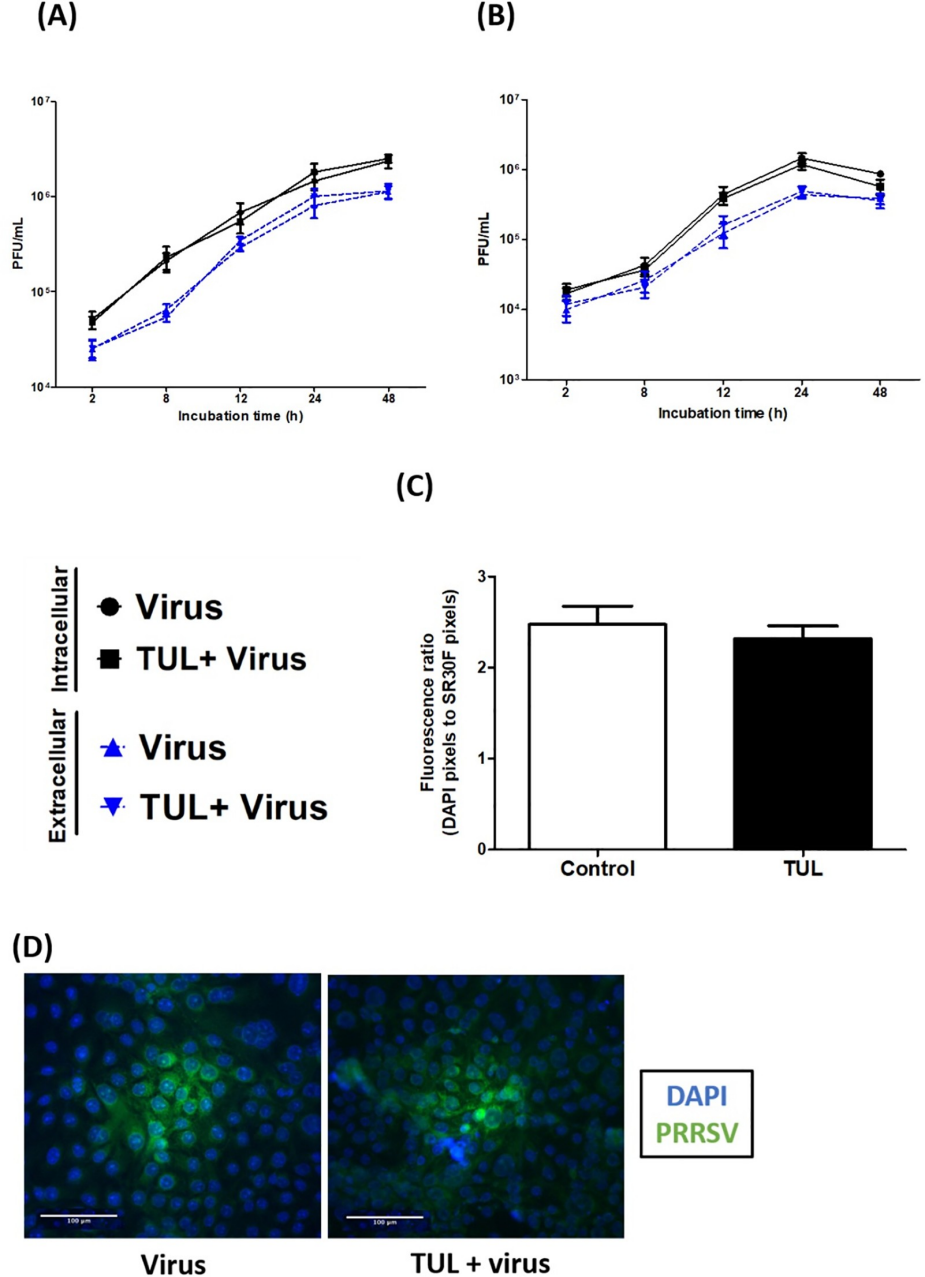
Tulathromycin does not alter PRRSV viral titers, inside or outside macrophages. Intracellular and extracellular infectious viral particles calculated in (A) MDMs or in (B) MARC-145, using plaque assays. MARC-145 were incubated with cell lysates (to detect intracellular viral particles; dashed line) or supernatants (to detect extracellular viral particles; full line). MDMs (A) or MARC-145 (B) were treated with TUL (1mg/mL) or not (Virus) for 1h, and infected with PRRSV (m.o.i of 0.1). (C and D) PRRSV-infected MARC-145 were stained at 24 hours p.i with a FITC-conjugated anti-PRRSV nucleocapsid SR30F antibody to allow the observation of intracellular PRRSV foci. MARC-145 nuclei were revealed using DAPI. (C) Fluorescence ratio (DAPI/SR30F) in double stained MARC-145 calculated using imageJ software. Fluorescence ratios, reflecting PRRSV infection, were not different between untreated cells and cells treated with tulathromycin. (D) Representative microscopic images of PRRSV foci (green) in MARC-145 infected with PRRSV 24h post-infection. MARC-145 nuclei are stained in blue (DAPI). Pictures are representative of 4 independent experiments. Bar = 100μm.

Another set of experiments assessed whether the effects of tulathromycin described above were associated with direct antiviral properties of the antibiotic, in porcine MDMs (Fig 13A) or MARC-145 cells (Fig 13B). Upon incubation with PRRSV, extracellular and intracellular viral particles were enumerated via plaque assay. Tulathromycin did not change intracellular or extracellular viral titers in either of the cell models (Fig 13A and 13B). To verify these results, MARC-145 cells were stained with FITC-conjugated anti-PRRSV nucleocapsid antibody SR30F antibody. Size and numbers of viral foci were calculated in presence or absence of tulathromycin (Fig 13C). Again, tulathromycin pre-treatment did not alter viral titers compared to exposure to PRRSV alone (Fig 13C and 13D).

### Tulathromycin does not change PRRSV receptor expression in MDMs

**Fig 14:**
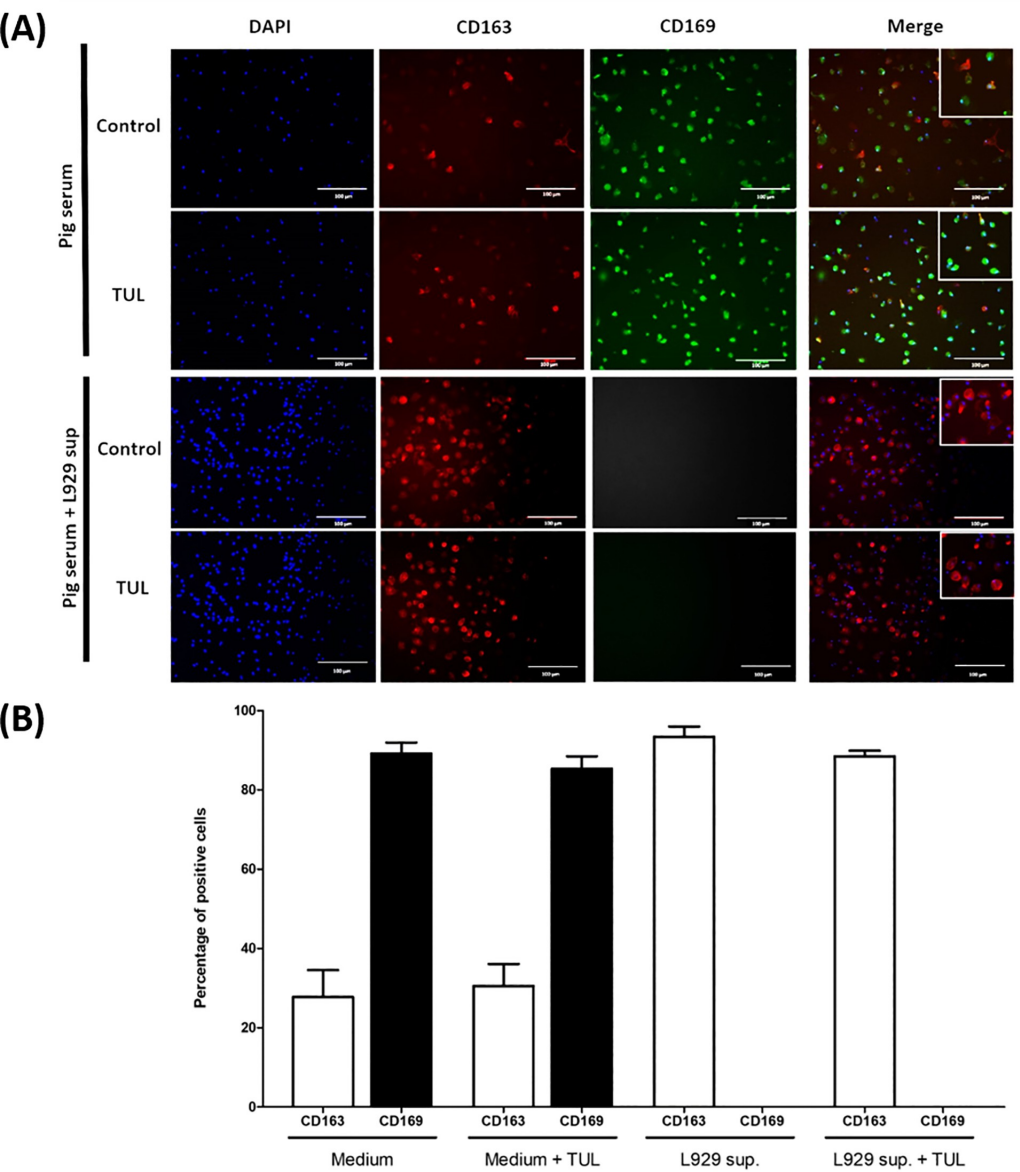
Tulathromycin does not change the experession of PRRSV receptors CD163 or CD169 in porcine MDMs. MDMs cultured in pig serum with or without L929 supernatant, and treated with HBSS (Control) or tulathromycin (TUL; 1mg/mL) for 12 hours. Cells were stained with CD163 (red) and CD169 (green) antibodies. (A) Fluorescent micrographs of MDMs stained for CD163 and CD169. Pictures are representative of 3 independent experiments. Bar = 100μm. (B) Percentage of MDMs, cultured with or without L929 supernatant, expressing CD163 or CD169. Treatment with TUL did not affect the expression of any of the receptors in cells cultured with or without L929. CD169 could not be detected in cells incubated with L929 supernant. n = 100 cells/group.

To further examine the effects of tulathromycin on PRRSV infectivity, experiments measured viral receptor expression in MDMs. To date, two major PRRSV receptors have been extensively studied (CD163 and CD169) and it is not entirely clear which one of these two receptors is essential for PRRSV infection [48–50]. Since L929-conditioned medium increases viral titers, we hypothesized that it might be due to an increase in cell permissivity resulting from an increase in PRRSV receptor expression. To test this hypothesis, we cultivated monocytes in medium containing pig serum alone or in L929-conditionned medium for 7 days and then treated them with tulathromycin. MDMs differentiated in medium devoid of L929-supernatant expressed both receptors. Approximatively 28% of cells expressed CD163 and 89% of cells expressed CD169. Tulathromycin treatment did not significantly change the percentage of CD163 and CD169 positive cells (respectively 29% and 83% of positive cells) (Fig 14A; upper panels; Fig 14B). MDMs incubation in L929-supernatant supplemented medium was sufficient to significantly increase the number of CD163 positive cells (more than 90% of MDMs were CD163 positive versus less than 30% in pig serum supplemented medium alone). In addition, following L929-supernantant exposure we were not able to detect any CD169 positive cells (Fig 14A; lower panels; Fig 14B). Tulathromcyin treatment following L929-incubation did not have any significant effect on viral receptor expression in these experiments (Fig 14A; lower panels; Fig 14B).

## Discussion

PRRS is one of the most devastating diseases of the porcine industry [1, 3]. Treatment options to control PRRS outbreaks are limited and the efficacy of vaccines is thwarted by the antigenic variability of PRRSV [51]. Disease severity is closely related to the ability of the virus to dysregulate macrophages functions and induce inflammation. Therefore, we hypothesize that targeting either of these components may represent a critical element of novel therapeutic approaches. Anti-inflammatory and immunomodulatory properties of macrolides have been well established [23–25–24]. Whether these effects may be beneficial in the context of viral diseases such as PRRS remains obscure. The present study assessed the anti-viral and immunomodulating properties of tulathromycin (TUL) in PRRSV–infected porcine macrophages. The findings indicate that TUL inhibits PRRSV-induced inflammatory responses in porcine monocyte-derived macrophages and protects against the phagocytic impairment caused by the virus, in the absence of any direct anti-viral effects.

The two most common cellular models are PAMs and MARC-145 cells [12, 52]. Both have significant limitations. The isolation of PAMs requires bronchoalveolar lavages, and the function of these cells depends on the age and environment of the animal [53]. Moreover, shortly after the initiation of a respiratory infection, alveolar macrophages are replaced by monocyte-derived macrophages, which therefore represent a key cell population in host- PRRSV interactions. Monkey MARC-145 epithelial cells do not originate from pigs. Therefore, the present experiments developed and used a simple porcine monocyte-derived macrophage model system to characterize the impact of tulathromycin on PRRSV infection. Previous *in vitro* studies have shown that the virus could infect blood monocyte-derived macrophages (MDMs) [54]. Consistent with previous findings, monocytes were less susceptible to PRRSV than differentiated monocyte-derived macrophages [54]. The addition of L929 supernatant during macrophage differentiation significantly increased their susceptibility to the virus compared to macrophages cultivated in medium supplemented with pig serum alone. It has been well established that L929 supernatant is a source of M-CSF and is used to induce macrophage differentiation [38]. Immunostaining of PRRSV receptors showed that the addition of L929 strongly upregulated CD163 (29% positive cells to 89% positive cells) but abolished CD169 expression. These results indicate that L929 supernatant modulate the expression of PRRSV receptors, and that CD163 alone is sufficient for PRRSV infection. This is consistent with recent observations showing that CD163, but not CD169, enabled non-permissive cells to become susceptible, and that increased CD163 correlates with increased susceptibility to PRRSV [49, 54, 55]. L929 supernatant is known to contain M-CSF, but very little is known about other cytokines and chemokines present in this supernatant [38]. Considering that CD163 and CD169 expression can be induced by IL-10 and IFN-γ respectively, and that IL-10 treatment increases PRRSV infectivity while IFN-γ decreases it [54, 56, 57], the role of these cytokine in the modulation of macrophage susceptibility to infection requires further investigation. The present findings demonstrate that MDMs can readily be infected by PRRSV, and hence represent a useful cellular model to study PRRSV pathogenesis, as suggested recently [37].

A hallmark of PRRSV pathogenesis resides in its ability to alter macrophages survival and function, hence predisposing the host to secondary infections [13, 58]. There is correlation between macrophage morphology and function, hence providing an easy way to monitor changes in macrophage polarization [41]. In this study we found that PRRSV infection dramatically altered monocyte-derived macrophage morphology, inducing an elongated phenotype with numerous cytoplasmic pseudopods. Recent findings indicate that these pseudopods promote intercellular junctions allowing PRRSV to evade host immunity through direct intercellular spread [59]. Tulathromycin pre-treatment was sufficient to prevent the PRRSV-induced pseudopod formation and morphological alterations in macrophages. Whether TUL may prevent intercellular junctions and thus hinder PRRSV immune evasion requires more research.

To test the hypothesis that change in macrophage morphology was associated with altered function, we measured the production of pro- and anti-inflammatory cytokines (CXCL-8 and IL-10 respectively) as well as the production of mitochondrial ROS. CXCL-8 is a potent neutrophil chemoattractant secreted by macrophages and other cell types, and is a critical mediator of neutrophil infiltration in inflamed tissues [26]. The present findings demonstrate that PRRSV is a potent inducer of CXCL-8 in monocyte-derived macrophages. Virally induced CXCL-8 secretion was inhibited by TUL. Studies in live animals are warranted to assess whether these observations suggest that TUL might attenuate PRRSV-induced inflammation through CXCL-8 inhibition. Mitochondrial ROS production is a hallmark of cell stress and inflammation, and contributes to PRRSV-induced tissue damage [42, 43]. Other reports showed that mitochondrial ROS production was implicated in PRRSV-induced apoptotic death of MARC-145 cells [43]. Here we demonstrate that PRRSV indeed induces ROS production in porcine monocyte-derived macrophages, and that this production is inhibited when the cells are pre-treated with the antibiotic. Tulathromycin was also able to restore ROS levels to control in LPS-stimulated cells but not in LPS and PRRSV exposed to both LPS and PRRSV. Interestingly, in these conditions MDMs showed a decrease in ROS production compared to cells exposed only to LPS. This suggest that PRRSV may inhibit intracellular ROS production of MDMs during bacterial infections.Another set of studies sought to determine whether TUL inhibition of the viral-induced pro-inflammatory CXCL-8 coincided with an increase in anti-inflammatory signaling. We found that the virus alone was able to inhibit IL-10 secretion, and that TUL blocked this effect. These data are in contrast with others from the scientific literature. Indeed, it is generally accepted that PRRSV induce IL-10 production to increase its infectivity [54]. In fact, IL-10 activated cells are more permissive to PRRSV than unstimulated M1-polarized macrophages [54]. More research is necessary to explain the mechanisms whereby PRRSV regulates the production of IL-10. Tulathromycin alone did not induce IL-10 secretion suggesting that CXCL-8 and mitochondrial ROS inhibition by TUL was not dependent on IL-10 production. Taken together the present findings strongly support the hypothesis that tulathromycin may attenuate PRRSV-induced inflammation by inhibiting production of pro-inflammatory CXCL-8, and by preventing the suppression of anti-inflammatory IL-10.

Consistent with previous studies, we found that PRRSV and TUL induced macrophage apoptosis [33, 43, 60, 61]. The present findings also illustrate that TUL and PRRSV haver additive pro-apoptotic effects. Morevoer, the data indicate that PRRSV leads to cell necrosis, an effect that was inhibited by TUL. Necrosis is known to exacerbate local inflammation, to induce the release of cytotoxic molecules, and to lead to extensive tissue damage, while cell apoptosis contributes to the resolution of inflammation [62, 63]. More research in live PRRSV-infected animals will help determine whether TUL is able to promote the resolution of PRRSV-induced pulmonary inflammation at least in part via such a mechanism, as well by shifting local cytokine release from pro-inflammatory to anti-inflammatory mediators.

It is well established that PRRSV infected pigs are often infected by secondary pathogens [13, 58]. At present, the mechanisms resulting in the increase of secondary infections during PRRSV infections remain incompletely understood. Studies have shown that PRRSV is directly able to impair macrophage phagocytosis, which in turn may represent a key element of the development of secondary infection [12, 64, 65]. Macrophage phagocytosis is triggered when phagocytic receptors including opsonic receptors (FcR) or pattern recognition receptors such as the mannose receptor, are activated [45, 66]. Using non-opsonized zymosan particles or IgG-coated latex beads, the present findings demonstrate that PRRSV significantly inhibits both phagocytic pathways. These results are consistent with previous reports showing decreased phagocytosis of latex beads, or live bacteria (*Streptococcus suis*) upon PRRSV infection [67, 68]. Recent findings suggest that PRRSV-1 inhibits phagocytosis through its interaction with sialoadhesin (also referred to as CD169). However, in our model system, L929 cultivated MDMs were negative for CD169 suggesting either that mechanisms for inhibition of phagocytosis are strain and/or genotype dependent, or that PRRSV may inhibit phagocytosis through multiple pathways [16]. Another report recently demonstrated that the same NSVL-98-7895 strain as used here may impair phagosomal maturation and NADPH oxidase-mediated respiratory burst, both implicated in the antimicrobial properties of macrophages [37]. TUL blocked the PRRSV-induced inhibition of non-opsonized and IgG-mediated macrophage phagocytosis. These observations pave the way towards studies *in vivo* to assess whether this antibiotic might help control secondary infections during PRRSV infections through this mechanisms in addition to its direct anti-microbial properties. The mechanisms whereby TUL protects against PRRSV-induced inhibition of phagocytosis require further elucidation.

Some macrolides such as tilmicosin and tylvalosin have been recently demonstrated to possess direct anti-viral effects against PRRSV [69, 70], while others like erythromycin do not [71]. In the experiments described herein, TUL did not exhibit any direct anti-viral properties, nor did it significantly alter the expression of the two receptors used by the virus for entry, CD169 and CD163. Together, the data indicate that in porcine MDMs, TUL is able to block PRRSV-induced pseudopod formation, necrosis, pro-inflammatory CXCL-8 and mitochondrial ROS production, and inhibition of macrophage phagocytosis. In addition TUL also synergized with PRRSV to induce pro-resolution cell apoptosis and the production of anti-inflammaotry IL-10. The results also show that the protective modulation of macrophage structure, function, and behavior by TUL occurs in the absence of a direct anti-viral effect. The present observations pave the way towards further studies with a PRRSV-1 strain to determine whether the effects we observed in this study are conserved with the other PRRSV genotype. Studies *in vivo* will help determine whether and how these effects may translate into clinical benefits.

## Supporting information

S1 Fig More than 95% of isolated cells express CD163. Flow cytometry analysis of CD163 expression on 7 days old MDMs. Data shown come from 3 independent experiments.
(DOCX)
