# Supplementary material for: Immuno-modulating properties of Tulathromycin in porcine monocyte-derived macrophages infected with porcine reproductive and respiratory syndrome virus
Source: PLoS One. 2019 Aug 23;14(8):e0221560. doi: 10.1371/journal.pone.0221560 (PMC6707645; doi:10.1371/journal.pone.0221560)
Supplement: S1 Fig — Flow cytometry analysis of CD163 expression on 7 days old MDMs. Data shown come from 3 independent experiments. (DOCX) [file pone.0221560.s001.docx]

**Supporting informations**


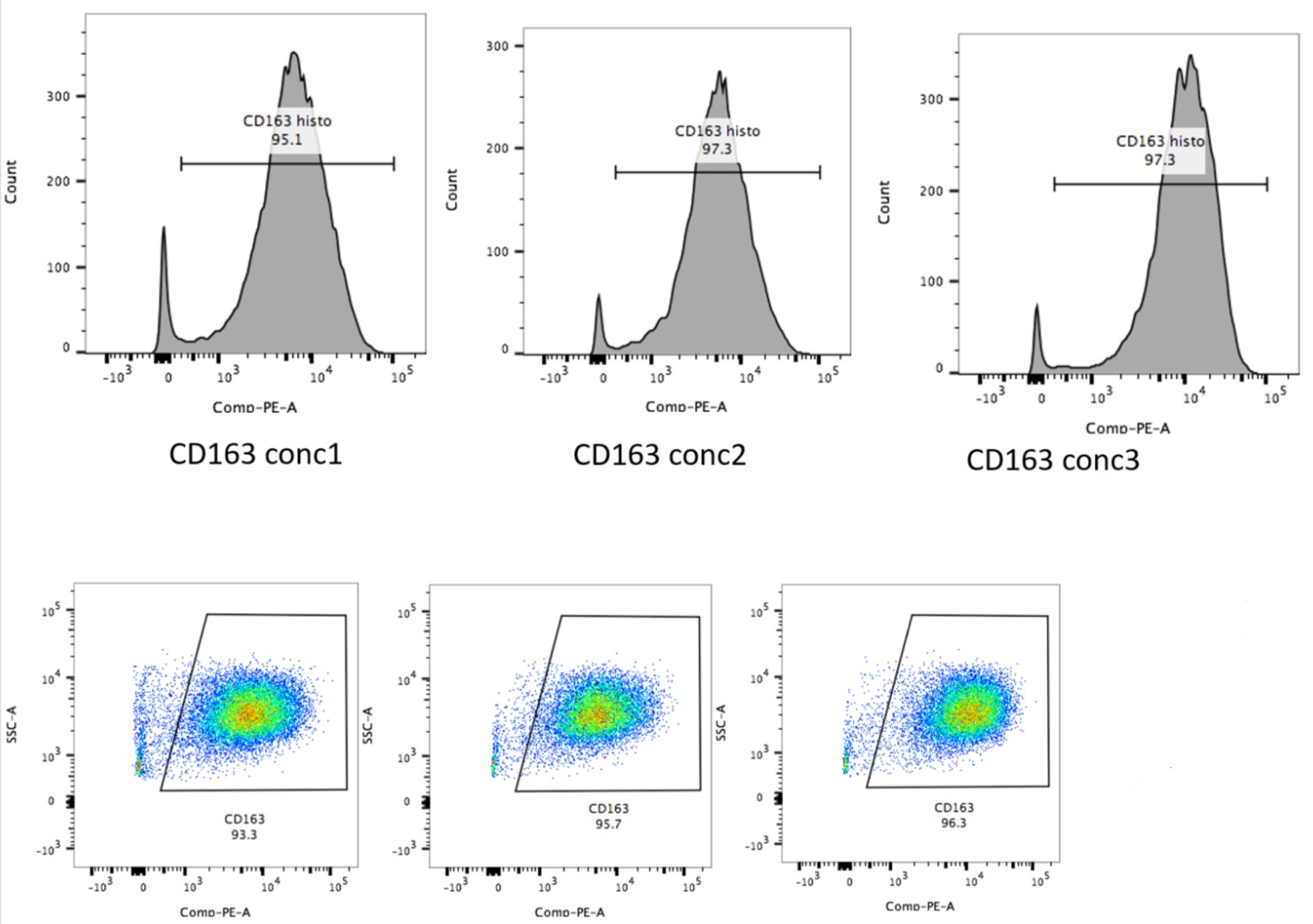


**S1 Fig. More than 95% of isolated cells express CD163**. Flow cytometry analysis of CD163 expression on 7 days old MDMs. Data shown come from 3 independent experiments.
